# Review of Domain Wall Dynamics Engineering in Magnetic Microwires

**DOI:** 10.3390/nano10122407

**Published:** 2020-12-01

**Authors:** Valentina Zhukova, Paula Corte-Leon, Lorena González-Legarreta, Ahmed Talaat, Juan Maria Blanco, Mihail Ipatov, Jesus Olivera, Arcady Zhukov

**Affiliations:** 1 Department Advanced Polymers and Materials: Physics, Chemistry and Technology, Faculty of Chemistry, University of Basque Country, UPV/EHU, 20018 San Sebastian, Spain; paula.corte@ehu.eus (P.C.-L.); lorena.glegarreta@gmail.com (L.G.-L.); aht17@pitt.edu (A.T.); mihail.ipatov@ehu.es (M.I.); 2 Department Applied Physics I, EIG, University of Basque Country, UPV/EHU, 20018 San Sebastian, Spain; juanmaria.blanco@ehu.es; 3 Department QUIPRE, Inorganic Chemistry-University of Cantabria, Nanomedice-IDIVAL, Avda. de Los Castros 46, 39005 Santander, Spain; 4 Department of Mechanical Engineering & Materials Science, Swanson School of Engineering, University of Pittsburgh, Pittsburgh, PA 15261, USA; 5 Nanoscience Research Laboratory, Pontificia Universidad Catolica Madre y Maestra, Autopista Duarte, Km 1 ½, 51000 Santiago, Dominican Republic; j.olivera@aduanas.gob.do; 6 Laboratorio de la Dirección General de Aduanas, Carlos Sánchez, Esquina Lope de Vega, Ensanche Naco, 10119 Santo Domingo, Dominican Republic; 7 IKERBASQUE, Basque Foundation for Science, 48011 Bilbao, Spain

**Keywords:** domain wall propagation, large Barkhausen jump, magnetic bistability, magnetic anisotropy, magnetostriction, magnetic microwire, internal stresses

## Abstract

The influence of magnetic anisotropy, post-processing conditions, and defects on the domain wall (DW) dynamics of amorphous and nanocrystalline Fe-, Ni-, and Co-rich microwires with spontaneous and annealing-induced magnetic bistability has been thoroughly analyzed, with an emphasis placed on the influence of magnetoelastic, induced and magnetocrystalline anisotropies. Minimizing magnetoelastic anisotropy, either by the selection of a chemical composition with a low magnetostriction coefficient or by heat treatment, is an appropriate route for DW dynamics optimization in magnetic microwires. Stress-annealing allows further improvement of DW velocity and hence is a promising method for optimization of DW dynamics in magnetic microwires. The origin of current-driven DW propagation in annealing-induced magnetic bistability is attributed to magnetostatic interaction of outer domain shell with transverse magnetization orientation and inner axially magnetized core. The beneficial influence of the stress-annealing on DW dynamics has been explained considering that it allows increasing of the volume of outer domain shell with transverse magnetization orientation at the expense of decreasing the radius of inner axially magnetized core. Such transverse magnetic anisotropy can similarly affect the DW dynamics as the applied transverse magnetic field and hence is beneficial for DW dynamics optimization. Stress-annealing allows designing the magnetic anisotropy distribution more favorable for the DW dynamics improvement. Results on DW dynamics in various families of nanocrystalline microwires are provided. The role of saturation magnetization on DW mobility improvement is discussed. The DW shape, its correlation with the magnetic anisotropy constant and the microwire diameter, as well as manipulation of the DW shape by induced magnetic anisotropy are discussed. The engineering of DW propagation through local stress-annealing and DW collision is demonstrated.

## 1. Introduction

Magnetic wires can present a rather unusual combination of exciting magnetic and transport properties, like magnetic bistability, giant magnetoimpedance (GMI) effect, giant magnetoresistance (GMR) effect, magnetic shape memory, and magnetocaloric effect [1,2,3,4,5,6,7]. Consequently, a number of prospective applications (magnetic memories, magnetic logics, magnetic sensors, magnetometers, magnetic tags, …) of magnetic wires have been reported [8,9,10,11,12,13,14].

One of the most promising phenomena, reported in various families of magnetic nano- and microwires, is the fast and controlled propagation of a single-domain wall (DW) [1,9,10,15]. Such DW propagation can be driven either by a magnetic field [15,16] or by an electric current [9,10,17].

DW propagation in ferromagnetic micro-nanowires is a subject of intense research, focusing on several problems, like the search for methods for reproducible DWs injection, controlling single or multiple DW propagation by electrical current, magnetic field, induced magnetic anisotropy or strain, controllable DW pinning, control the DW structure through the geometrical dimensions among others [1,10,18,19,20,21,22,23].

Several of the aforementioned applications (racetrack memories, magnetic logics, magnetic and magnetoelastic sensors, magnetic tags) involve fast magnetization switching and controllable DW propagation [9,10,14,18,19,20]. Most of these applications present advanced features. Thus, magnetic logic based on DW propagation has several advantages over conventional electronic logic, for example, it heats up very little during data switching due to the lack of transistors.

In most of the publications, DW propagation in cylindrical amorphous micrometric and submicrometric wires DW velocities, *v*, well above 1 km/s have been reported [15,16,17,18,21,22,24]. Such high DW velocities have been observed even in as-prepared microwires [15,16,17,18,24]. However, such elevated *v*-values can be further considerably improved (up to 3–4 km/s) either by appropriate annealing [25,26] or by a transverse magnetic field or transverse magnetic anisotropy induced by specially designed thermal treatment [19,20,27,28].

Such extremely fast DW dynamics, observed in magnetic microwires, is rather unusual: similar DW velocities and phenomena related to such elevated magnitude of DW velocity (i.e., Cherenkov emission of sound by a moving DW or magnetoelastic interaction of resonant character with the acoustic subsystem of the crystal) have been previously reported only for weak ferromagnets [29,30].

The origin of such elevated DW velocity in amorphous micrometric and submicrometric wires, understanding of the factors limiting the DW velocity as well as searching of the routes allowing further DW velocities improvement is therefore essentially relevant for the implementation of various applications involving DW propagation in different magnetic wire families.

Amorphous or nanocrystalline magnetic microwires in which quasi-supersonic DW velocities have been reported can be prepared using the Taylor–Ulitovsky technique involving rapid solidification from the melt of ferromagnetic metallic nucleus inside the glass coating [24,31,32]. It is worth mentioning, that magnetic bistability and single DW propagation have been reported in thicker amorphous wires (typically of 120 μm in diameter) prepared by the in-rotating water method, however, *v*-values reported for this family of thicker wires are typically much below 1 km/s (i.e., an order of magnitude lower) [33,34,35]. The aforementioned Taylor–Ulitovsky preparation technique allows 1–2 orders of magnitude wire diameter extension toward submicrometric diameters range [24,31,32,36,37]. The micro-submicrometric wires prepared using the Taylor–Ulitovsky technique consist of a perfectly cylindrical metallic amorphous nucleus surrounded by flexible insulating glass coating [31,32,33]. The preparation method is described in detail in refs. [29,30] was developed in the 1960s initially for the preparation of non-magnetic microwires [38,39].

A flexible and insulating glass coating is an integral part of the preparation process, providing functional properties such as reduced dimensionality, improved corrosion resistance, and in some cases mechanical properties [40,41,42,43]. However, simultaneous solidification of metallic alloy and glass coating with rather different thermal expansion coefficients results in the arising of internal stresses [44,45]. Therefore, magnetoelastic anisotropy together with shape anisotropy and defects related to preparation and post-processing techniques are the main factors that affect the magnetic properties of magnetic microwires.

Thus, amorphous magnetic microwires with positive magnetostriction coefficient generally present spontaneous magnetic bistability originated by a single and large Barkhausen jump between two remanent states with opposite magnetization: the demagnetized state cannot be observed in magnetically bistable microwires. Such magnetically bistable microwires, therefore, present perfectly rectangular hysteresis loops, and the magnetization switching (between two remanent states) runs by fast DW propagation. As reported elsewhere [5,15,22,25], such DW propagation starts from the microwire ends, where the closure domains exist because of the demagnetizing field effect.

Accordingly, if the DW propagation from one end of the microwire is hindered (for example, by a lower applied magnetic field) the single DW propagation can be realized in magnetically bistable microwires [16,18,46,47]. Thus, magnetically bistable microwires are a unique material allowing studies of single DW propagation.

Spontaneous magnetic bistability can be observed in as-prepared amorphous [16,21,46,47] and nanocrystalline [48,49] microwires with positive magnetostriction coefficient, it can be maintained after devitrification of amorphous microwires [49,50] and even it can be induced by appropriate annealing in amorphous microwires with low negative magnetostriction coefficient [26]. In the latter case, current-induced single DW propagation has been observed [17].

Insulating non-magnetic glass coating is the origin of strong internal stresses. In fact, the difference in the thermal expansion coefficients of metallic alloy nucleus solidifying simultaneously with the glass coating surrounding is the main source of internal stresses [32,43,44,51]. Therefore, the magnitude of internal stresses inside the metallic nucleus can be tuned by the *ρ*-ratio between the metallic nucleus diameter, d, and the total microwire diameter, D (*ρ* = d/D) [32,43,44,51,52].

Tensile and torsion stresses can be easily applied to microwires, allowing one to study the applied stress influence in single DW dynamics [21,53]. In addition, a single DW can be injected either by a local magnetic field or by artificially created defects allowing the DW nucleation [54,55,56]. Accordingly, trapping, collision, or injection of DWs can be used for the manipulation of DW dynamics in microwires [54,55,56,57].

In this way, magnetic microwires provide a unique possibility to study the effect of various factors, like applied or internal stresses, magnetostriction coefficient, magnetoelastic and induced magnetic anisotropy, transverse and local magnetic field on single DW propagation.

Therefore, the purpose of this paper is to review the extensive studies on DW dynamics to evaluate the routes for optimization of DW dynamics in magnetically bistable microwires.

## 2. Materials and Methods

All studied magnetic microwires have been prepared using the Taylor-Ulitovsky technique described elsewhere in detail [31,32]. Briefly, the microwire preparation consists of melting of the previously prepared metallic alloy inside a glass tube by a high-frequency inductor heater, forming a glass capillary, and finally rapid solidification of the composite wires (cylindrical metallic nucleus inside the glass coating). Prepared in this way, microwires can have metallic nucleus diameters ranging from 0.05 to 100 μm covered by thin (typically of 0.5–20 μm in thickness), flexible and insulating glass coating [31,32,38,39]. The microwires compositions and geometrical characteristics are provided in Table 1.

The DW dynamics have been analyzed in as-prepared and annealed microwires. X-ray Diffraction (XRD) using a BRUKER (D8 Advance) X-ray diffractometer with Cu K_α_ (*λ* = 1.54 Å) radiation and Differential Scanning Calorimetry, DSC, measurements using DSC 204 F1 Netzsch calorimeter in Ar atmosphere at a heating rate of 10 K/min have been employed to control the structure of as-prepared and annealed samples.

Heat treatment was carried out in a conventional furnace. The annealing temperature, *T_ann_*, was chosen between 200 °C and 700 °C, and the annealing time, *t_ann_*, was up to 150 min. In some cases, we employed annealing under tensile stress. Stress-annealing is performed as follows: the sample is first loaded with stress (attaching a mechanical load to the microwire end), then placed in a pre-heated furnace until the temperature is established. Finally, it was slowly cooled in the air while applying stress in the furnace. Thus, tensile stress was applied both, during annealing and cooling of the sample in the furnace. The magnitude of the stress during the annealing within the metallic nucleus was estimated as described earlier [56]:

(1) $$\sigma_{m}=\frac{K\cdot P}{\;K\;\cdot S_{m}{+\;S}_{gl}}$$

where *K = E*_2_/*E*_1_, *E*_2_, and *E*_1_ are the Young’s moduli of the metal and the glass, respectively, *P* is the applied mechanical load, *S_m_* and *S_gl_* are the cross-sections of the metallic nucleus and glass coating, respectively. The stress magnitude evaluated by Equation (1) was up to 900 MPa.

The hysteresis loops were measured using the fluxmetric method described earlier [58]. For a better comparison of samples with different chemical compositions and annealed at different conditions, we present the hysteresis loops as the dependence of the normalized magnetization M/M_0_ (being M—the magnetic moment at a given magnetic field, and M_0_—the magnetic moment of the sample at the maximum magnetic field amplitude) versus the magnetic field, H.

The magnetostriction coefficient of the microwires was measured using the small-angle magnetization rotation (SAMR) method [59]. We have used a recently developed experimental setup adapted for thin microwires [60].

**Figure 1:**
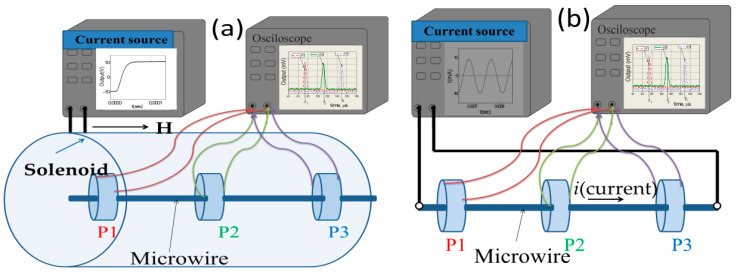
Schematic picture of the experimental set-up for measurements of magnetic field driven (**a**) and current driven (**b**) DW dynamics in microwires.

We measured the magnetic field dependence of DW velocity, *v*, of a single DW traveling along with the sample by a modified Sixtus–Tonks method previously described elsewhere [19,35]. The principal difference of the used method from the classical Sixtus–Tonks [61] set-up is the following: one sample end is placed outside the magnetization coil to ensure a single DW propagation. We employed three pick-up coils to avoid multiple DW propagation and hence overestimating the magnitude of *v* due to multiple DW propagation (see Figure 1a) [15,35,36,53,56].

For studies of the current-driven DW dynamics instead of a magnetizing solenoid, we used the AC flowing through the microwire (see Figure 1b).

Then, *v* can be evaluated as

(2) $$v\;=\;\frac{l}{\Delta t}$$

where *l* is the distance between pick-up coils and Δ*t* is the time difference between the electromotive force (EMF) peaks originated from moving DW in the pick-up coils [15,16,17].

For the DW injection inside the sample and nucleation field profile evaluation, we used the set-up allowing to apply local magnetic field by a short magnetizing coil [54]. This short magnetizing coil is located next to the short pick-up coil, which allows local magnetization reversal to be detected at a sufficiently large distance from the ends of the wire. Then the microwire was slowly moved through this magnetizing coil, which made it possible to measure the length, *L*, distribution of the local magnetization reversal (DW injection) fields of each sample.

The microscope Axio Scope A1 was used for the defects evaluation in magnetic microwires.

## 3. Results and Discussion

- Single and multiple DW propagation regimes: defect influence and limits of a single domain wall regime.

There are several families of magnetic microwires with single DW propagation. They can have different (amorphous or nanocrystalline) structure. In addition, amorphous (based on Fe and Fe-Ni) microwires with a positive magnetostriction coefficient, *λ_s_*, exhibit spontaneous magnetic bistability, and, therefore, DW propagation can be observed even in as-prepared microwires. However, magnetic microwires with vanishing *λ_s_* (Co-rich compositions) exhibit annealing-induced magnetic bistability.

**Figure 2:**
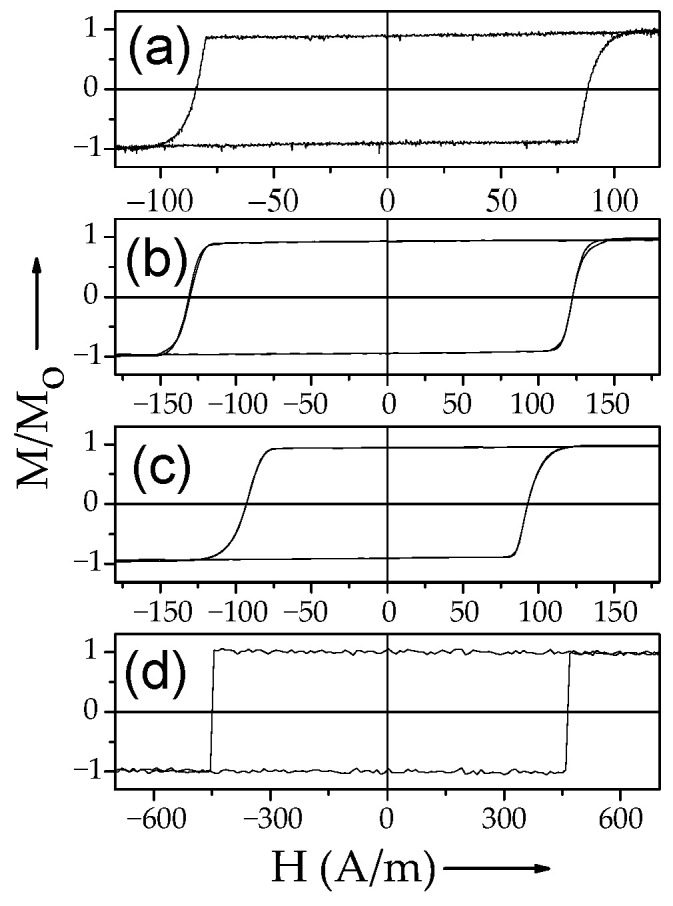
Hysteresis loop of as-prepared amorphous Fe_75_B_9_Si_12_C_4_ (**a**), Fe_62_Ni_15.5_Si_7.5_B_15_ (**b**), annealed at 300 °C (1 h) Fe_3.6_Co_69.2_Ni_1_B_12.5_Si_11_Mo_1.5_C_1.2_ (**c**), and as-prepared nanocrystalline Fe_38.5_Co_38.5_B_18_Mo_4_Cu_1_ (**d**) microwires.

Several examples of hysteresis loops of amorphous and nanocrystalline microwires exhibiting spontaneous (Fe_75_B_9_Si_12_C_4_ and Fe_62_Ni_15.5_Si_7.5_B_15_) and annealing-induced (Fe_3.6_Co_69.2_Ni_1_B_12.5_Si_11_Mo_1.5_C_1.2_) magnetic bistability are shown in Figure 2a–d.

Accordingly, below we will describe the main common and specific features of DW propagation in each family of magnetic microwires.

### 3.1. Single DW Propagation in Amorphous Microwires and Role of Defects

First experimental results on magnetic-field-driven DW dynamics in amorphous magnetic microwires were reported almost 20 years ago [46,62]. The main point observed in the first papers on DW propagation was that the DW, nucleated by the nucleation coil, can travel even if the applied magnetic field, H, is below the switching field. Non-linear *v*(*H*) dependencies observed for low field region can be attributed to thermally activated DW motion [63].

Roughly linear *v*(*H*) dependencies, reported in subsequent publications on DW dynamics in amorphous microwires [15,47], can be well understood in terms of the viscous DW motion [63]. However, elevated values of *v* (generally above 1 km/s) obtained for amorphous microwires [15,16,47] were clearly superior to magnitudes of *v* reported for thicker amorphous and crystalline wires [33,34,35,64].

As described elsewhere [15,16,17,18,33,34,35], in a viscous regime, the DW propagates with a velocity, *v*, given as

*v* = *S* (*H* − *H*_0_) (3)

where *S* is the DW mobility, *H* is the axial magnetic field and *H*_0_ is the critical propagation field.

On the other hand, essentially non-linear *v*(*H*) dependencies and even supersonic magnitudes of *v* have been reported for magnetic microwires [16,65]. Initially, deviations from linear *v*(*H*) dependencies have been attributed to either elastic waves generated by DWs and interaction of the domain wall with phonons or Walker-like behavior [66]. However, later, a simpler interpretation has been proposed based on a comparison of *v*(*H*) dependencies and distribution of local nucleation fields along the microwires [15,67,68].

Thus, experimentally, it was observed [54], that the DW can be injected by a high enough magnetic field in any part of the microwire.

**Figure 3:**
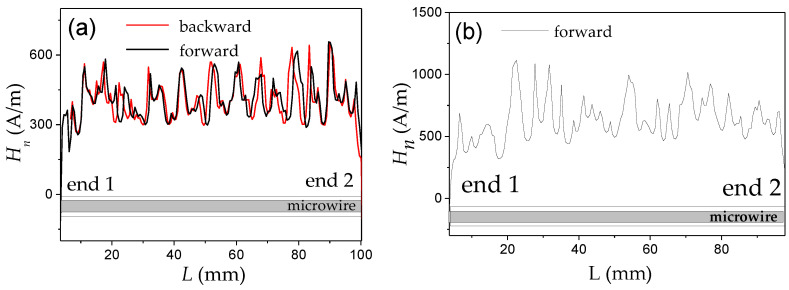
Distribution of the local nucleation fields, *H_n_*(*L*), measured in two different samples of magnetically bistable amorphous Fe_74_B_13_Si_11_C_2_ microwire. In (**a**) *H_n_*(*L*) dependence is measured in two directions along the sample and (**b**) *H_n_*(*L*) dependence is measured in the forward direction along the sample. Reprinted with permission from [68,69], Copyright Eslevier, 2014, 2012.

Two examples of the distribution of local nucleation fields, *H_n_*, measured in two different samples of amorphous Fe_74_B_13_Si_11_C_2_ microwires are shown in Figure 3. Although both samples have the same dimensions (*d* and *D*), the *H_n_*(*L*) dependencies are different. Specifically, the evolution of *H_n_* along the length, *L*, and the magnitude of the *H_n_* oscillations are different. As discussed elsewhere [54,67,68], the *H_n_*(*L*) oscillations must be attributed to defects.

The main features of the *H_n_*(*L*) distributions are the following:

(i) The *H_n_*(*L*) dependencies, measured by moving the same microwire forward and backward through the magnetizing coil, have the same oscillations sequence and amplitude (see Figure 3a). Accordingly, *H_n_*(*L*) distributions are completely reproducible for a given sample (see Figure 3a). In some sense, *H_n_*(*L*) distribution is the signature of each sample. A slight shift in the *H_n_*(*L*) dependencies must be attributed to the precision of the forward and backward microwire movement.
(ii) In all *H_n_*(*L*) distributions *H_n_* values near the sample ends are considerably lower than in the middle part of each sample.

From the above presented *H_n_*(*L*) distributions, we can assume that if the external field is above the minimum nucleation field in the middle portion of the given sample, several DWs can propagate within the sample: DW depinned from the sample ends as well as DWs injected in the middle part of the sample. Consequently, single DW propagation can be realized only in the determined magnetic field range: if the applied magnetic field is below the minimum nucleation field in the middle part of the microwires. This mechanism has been confirmed several times [67,68,69,70] through the correlation between the *v*(*H*) dependencies and *H_n_*(*L*) distributions.

**Figure 4:**
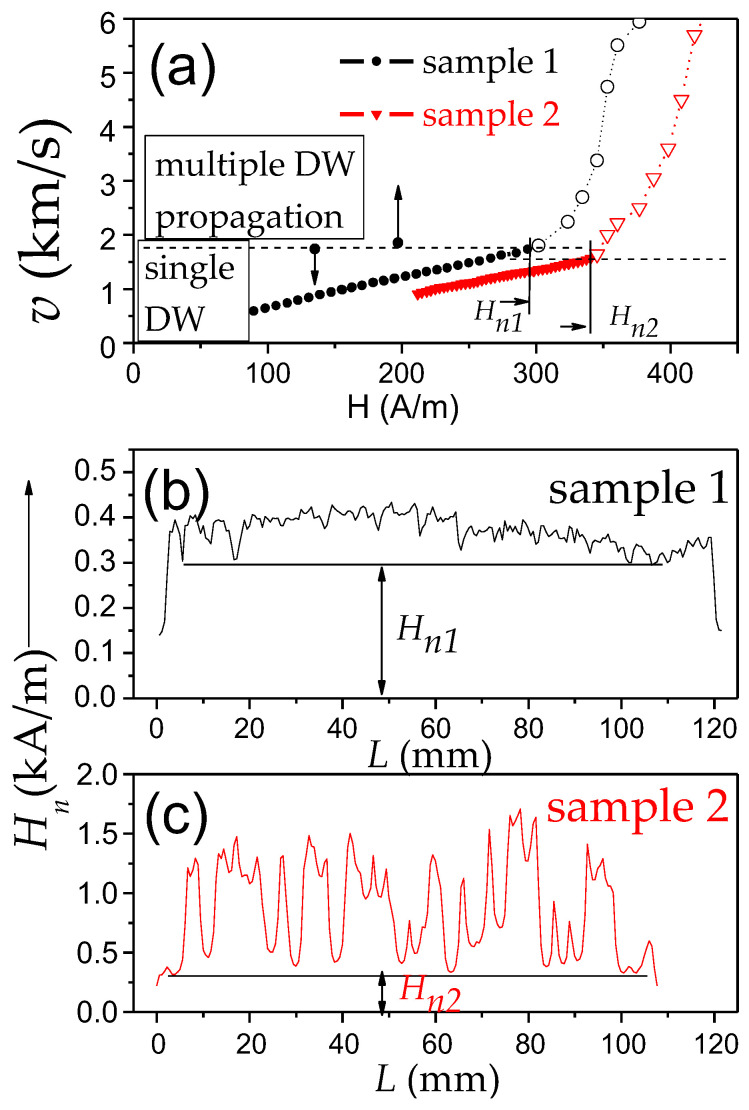
Dependence of DW velocity, *v*, on the magnetic field, H, measured on magnetically bistable amorphous Fe_74_Si_11_B_13_C_2_ (sample 1) and Fe_75_Si_12_B_9_C_4_ (sample 2) microwires (**a**) and distribution of local nucleation fields measured in the same samples (**b**,**c**). Reprinted with permission from ref. [67].

An example of such correlation is shown in Figure 4, where *v*(*H*) depends on two bistable amorphous glass-coated microwires Fe_74_Si_11_B_13_C_2_ and Fe_75_B_9_Si_12_C_4_, (*ρ*-ratios 0.85 and 0.76, respectively) marked as sample 1 and 2, respectively, are presented. In both samples, linear *v*(*H*) dependencies are observed up to H = 294 and 340 A/m for samples 1 and 2, respectively (maximum DW velocity *v* of 1.7 km/s). However, for the region of higher H, deviations from the linear *v*(*H*) dependencies are clearly seen (Figure 4a).

The overall minimum, *H_n_*_1_ and *H_n_*_2_, observed in the *H_n_*(*L*) distributions for both microwires correlate quite well with the onset fields of deviation from linear *v*(*H*) dependencies (see Figure 4b,c).

In the *H_n_*(*L*) dependencies of both studied samples, we can observe oscillations that have been attributed to the positions of the defects. The higher oscillations *H_n_*(*L*) amplitude in sample 2 must be attributed to the higher defect content and efficiency.

Considering that at H ≥ *H_n_*_1_ and H ≥ *H_n_*_2_ a new domain can be injected into the microwire, we attributed the observed correlation of deviations from linear *v*(*H*) dependencies and *H_n_*_1_ and *H_n_*_2_-values as a change between single and multiple DW propagation regimes.

Accordingly, neglecting new domain nucleation at a high enough magnetic field, H, above minimum nucleation field, *H_nmin_*, can result in exaggerated magnitudes of *v* from Sixtus–Tonks experiment. Therefore, reliable results on DW dynamics and correct values of *v* can be evaluated for H ≤ *H_nmin_* for a given microwire sample. If H ≥ *H_nmin_*, several DWs (one from the wire end and others from the reversed domains nucleated in the central part of the microwire) can propagate simultaneously. Thus, two pick-up coils Sixtus–Tonks-like set-up [16,65] has been modified by us to obtain reliable and correct *v*-values. To detect the possible nucleation and subsequent propagation of several DWs, we have applied the three pick-up coils setup, described in the Section 2.

**Figure 5:**
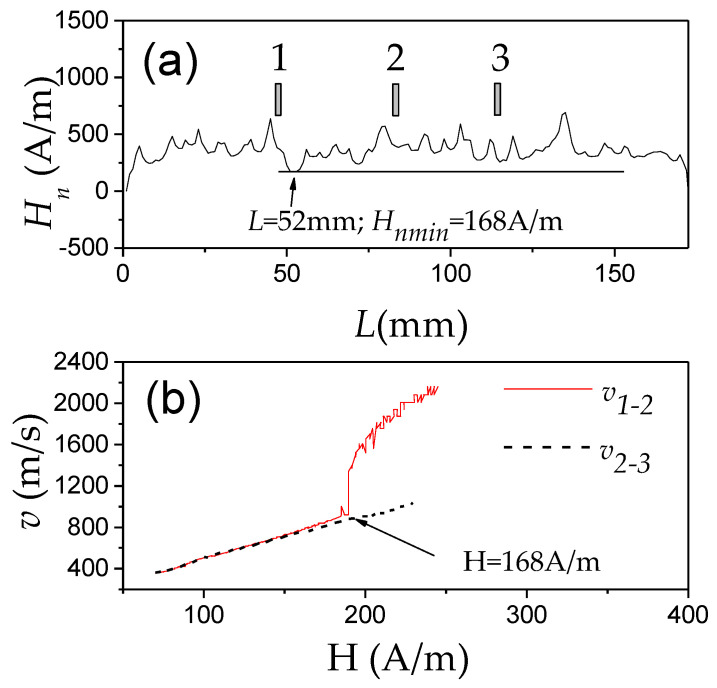
Correlation of local nucleation field distribution (**a**) and dependencies of DW velocity, *v*, on the magnetic field, H, (**b**) measured in magnetically bistable amorphous Fe_74_B_13_Si_11_C_2_ microwire. 1, 2, 3 are the positions of the pick-up coils. Reprinted with permission from ref. [15].

Correlation of the deviation from linear *v*(*H*) dependencies and *H_n_*(*L*) distribution, related to the change between the single and multiple DW propagation regimes, have been observed even in different portions of the same sample [15,68,69,70]. An example is provided in Figure 5, where the *H_n_*(*L*) distribution (Figure 5a) and *v*(*H*) dependencies (Figure 5b) measured between coils 1–2 and 2–3, *v*_1–2_(*H*) and *v*_2–3_ (*H*), respectively, are compared. As can be observed, when H ≥ *H_nmin_* (for coils pair 1–2 *H_nmin_* ≈ 168 A/m at *L* = 52 mm), the abrupt increase in *v*_1–2_ is observed. At the same time, *v*_2–3_ did not show any jump on *v*_2–3_ (*H*) dependence in this field region (Figure 5b). Consequently, we assume that the jump observed in *v*_1–2_(*H*) dependence at *H* ≈ 168 A/m must be attributed to new DW injection.

Similar correlations of *v*(*H*) dependencies and *H_n_*(*L*) distribution have been reported for various samples: correlation of *H_nmin_*-value and deviation from linear *v*(*H*) dependencies at H ≈ *H_nmin_* has been observed elsewhere [15,68,69,70].

Moreover, a simple model allowing to evaluate the position of the DW nucleation with respect to the pick-up coil position from the *H_n_*(*L*) distribution has been proposed [69].

Accordingly, the main features of a single DW propagation regime in magnetic microwires can be summarized as follows:

(i) Single DW propagation in the viscous regime can be observed in the magnetic field range between the switching field, *H_s_*, and *H_nmin_*, determined from the *H_n_*(*L*) distribution. Therefore, the extension of a single DW regime is influenced by the factors affecting *H_nmin_* and *H_s_*-value.
(ii) *H_s_* is determined as the magnetic field at which the DW depinning from the wire end takes place. Generally, the switching field magnitude is affected by the magnetoelastic and shape anisotropies.
(iii) *H_nmin_* is limited by the defects. Therefore, the extension of the linear *v*(*H*) dependence is determined by the defects and their distribution.

**Figure 6:**
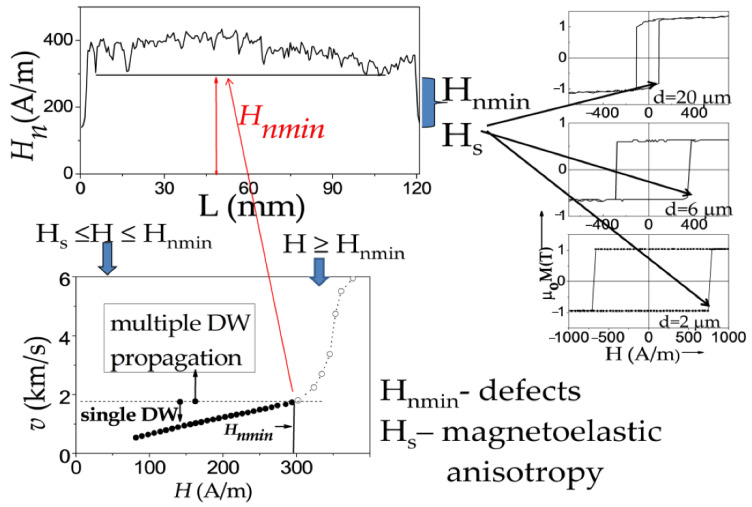
Schematic picture illustrating the factors affecting the single DW propagation regime in magnetic microwires.

These features are summarized in Figure 6.

It is worth noting, that the defects can lead to a considerable acceleration of the magnetization switching. Indeed, if the applied magnetic field is high enough (above *H_nmin_*), a new reversed domain can be injected in front of the propagating DW. Consequently, faster magnetization switching can be observed.

On the other hand, one of the ways to obtain higher single DW velocities is to diminish the content of defects by the preparation technology improvement.

It is known that in conventional magnetic materials different types of defects (dislocations, impurities, edge and surface roughness, etc.) can induce unwanted pinning of DW, thus changing the performances of the devices based on them. In amorphous materials the defects typical for crystalline materials (grain boundaries, dislocations, twins…) are absent. Accordingly, the following factors affecting the magnetic softness of amorphous materials are identified and discussed by H. Kronmüller [71]:

1. Intrinsic fluctuations of exchange energies and local anisotropies,
2. Clusters and chemical short ordered regions,
3. Surface irregularities,
4. Relaxation effects due to local structural rearrangements,
5. Volume pinning of domain walls in magnetostrictive alloys,

Accordingly, understanding the origin of the defects determining the *H_nmin_* and *H_s_* values, and limiting the DW velocity is essentially important.

The magnetostriction contribution is considered as one of the most relevant terms affecting the coercivity of amorphous materials. In the case of glass-coated microwires, the contribution coming from the magnetoelastic anisotropy is even more relevant [44,45].

The origin of defects and their correlation with the DW dynamics are analyzed in several publications [71,72,73,74].

The most typical kind of defect observed by metallographic methods is the presence of bubbles inside the glass coating forming during the fabrication process of the microwire [71,72,73]. These bubbles have been observed in Fe and Co-rich microwires.

The other kinds of defects are glass thickness inhomogeneities, the interfacial layer between the metallic nucleus and the glass coating, and the oxides [71,72,73]. The defects distribution has a spontaneous character (qualitatively similar to the *H_n_*(*L*) distribution) [71]. The most common defects (bubbles) can potentially serve as stress inhomogeneities spontaneously distributed along the length of the sample.

**Figure 7:**
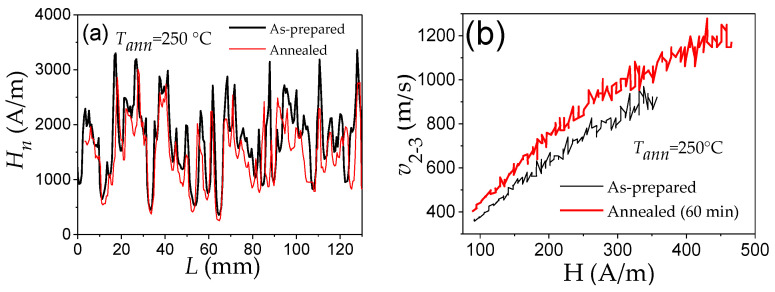
Local nucleation field distributions, *H_n_*(*L*), (**a**) and dependencies of DW velocity, *v*, on the magnetic field, H, (**b**) of as-prepared and annealed Fe_66.7_Cr_11.4_B_12_Si_9_Ni_0.9_ microwire. Adapted from ref. [70].

The most common way to reduce the stresses inhomogeneity is the thermal treatment. Accordingly, the experimental results on the effect of annealing on the *H_n_*(*L*) distribution (see Figure 7a) and on the *v*(*H*) dependence (Figure 7b) confirm the important contribution of the internal stresses inhomogeneity: the amplitude of oscillations of local nucleation fields decreases, while the expansion of *v*(*H*) dependences (and hence, the maximum *v* magnitude) increase after annealing. Both dependencies could be attributed to stress relaxation after annealing.

Consequently, appropriate annealing is a potentially suitable method for tuning DW dynamics in magnetic microwires.

Below, we will provide several routes for optimization of the DW dynamics in magnetic microwires with either spontaneous or annealing-induced magnetic bistability. We will pay attention only to the linear *v*(*H*) dependencies corresponding to a single DW propagation in the viscous regime, without touching the non-linear *v*(*H*) dependencies at the high field region.

#### 3.1.1. DW Propagation in Amorphous Microwires with Spontaneous Magnetic Bistability

- DW propagation in as-prepared amorphous microwires
- Optimization of DW dynamics by annealing
- Effect of induced magnetic anisotropy on DW dynamics
- Manipulation of DW dynamics

As mentioned above, spontaneous magnetic bistability can be observed in as-prepared amorphous microwires with a positive magnetostriction coefficient, *λ_s_*. Such microwires present a perfectly rectangular hysteresis loop [5,15]. The magnetostriction coefficient, *λ_s_*, of amorphous alloys are affected by the chemical composition [60,75,76,77,78,79,80]. Generally, Fe-rich amorphous alloys have positive *λ_s_* with maximum of about *λ_s_*~40 × 10^−6^, reported for (Co_x_Fe_1 − x_)_75_M_25_ (M = B, Si, C, P) alloys at x ≈ 0.2 [60,75,76,81,82,83]. In Co-rich amorphous alloys low and negative *λ_s_* up to *λ_s_*~−5 × 10^−6^ are reported [60,75,76]. Accordingly, nearly-zero *λ_s_* can be obtained in the Co_x_Fe_1 − x_ (0 ≤x ≤ 1) or Co_x_Mn_1 − x_ (0 ≤ x ≤ 1) alloys for 0.9 ≤ x ≤ 0.96 [60,75,76,77,84,85]. Similarly, a decrease in *λ_s_* is reported for Ni_x_Fe_1 − x_ (0 ≤ x ≤ 1) amorphous alloys rising Ni content. However, Ni-based amorphous alloys are not ferromagnetic at room temperature [76].

**Figure 8:**
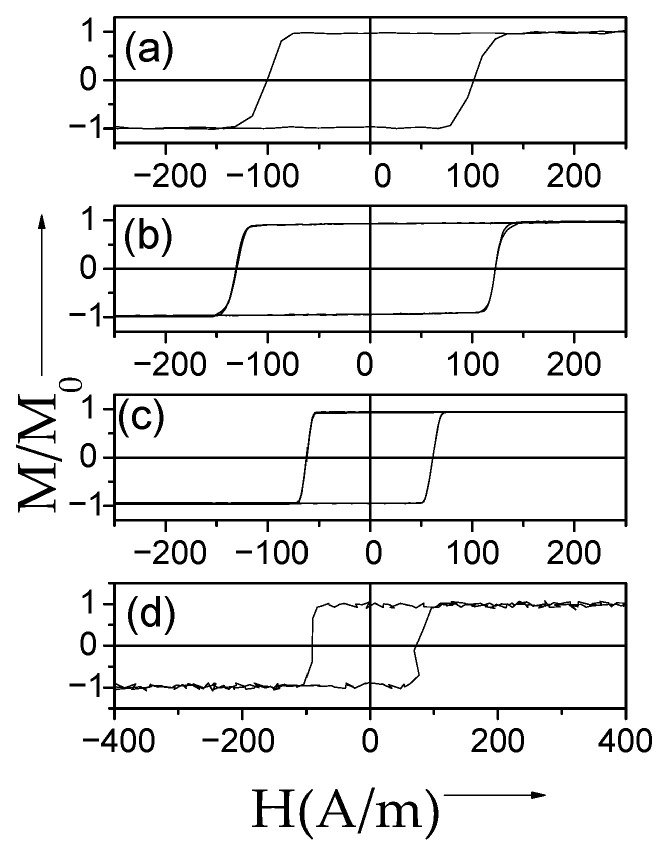
Hysteresis loops of as-prepared Fe_77.5_Si_7.5_B_15_ (**a**), Fe_62_Ni_15.5_Si_7.5_B_15_ (**b**), Fe_47.4_Ni_26.6_Si_11_B_13_C_2_ (**c**), and Fe_16_Co_60_Si_13_B_11_ (**d**) microwires. Adapted from refs. [83,86].

Consequently, spontaneous magnetic bistability characterized by perfectly rectangular hysteresis loops can be observed in various Fe-rich, Co_x_Fe_1 − x_, and Ni_x_Fe_1 − x_ amorphous microwires (see Figure 8).

As shown elsewhere [17,26], the hysteresis loops of as-prepared amorphous microwires with negative *λ_s_* are rather different: for such microwires linear and almost non-hysteretic loops with low coercivity, *H_c_*, are observed.

This difference is commonly explained by the decisive contribution of the magnetoelastic anisotropy, *K_me_*, given by [15,17,44,45,51]:

*K_me_* = 3/2 *λ_s_σ*, (4)

where *σ*_=_ *σ_i_*_+_ *σ_a_*_,_ *σ_i_* and *σ_a_* are the internal and applied stresses, respectively.

If the *λ_s_* value can be easily modified by the selection of different chemical compositions, *σ_i_* is determined by the preparation method. Up to now, the following sources of the internal stresses have been identified: (i) different thermal expansion coefficients of the metallic alloy and the glass coating; (ii) rapid melt quenching stresses; and (iii) the drawing [44,45,51]. The main contribution in total internal stresses is due to the difference in the thermal expansion coefficients of the metallic alloy and the glass coating, being an order of magnitude larger than the other two contributions [44,45,51]. Accordingly, most of the experimental results point out the correlation between *σ_i_* and *ρ* [44,45,51,52].

Consequently, one can expect that both, *λ_s_* and *σ_i_*,-values, can affect the DW dynamics. In addition, to clarify the effect of magnetoelastic anisotropy, it is reasonable to fix one of the parameters and modify the other.

Below, we present several experimental pieces of evidence of the effect of magnetoelastic anisotropy on DW dynamics.

**Figure 9:**
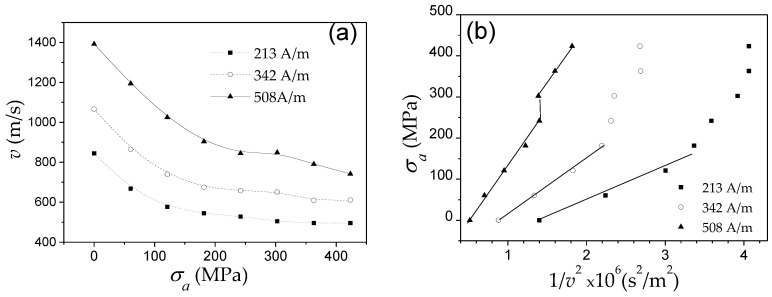
Dependencies of DW velocity, *v*, on applied stress, *σ_a_*, of Fe_55_Co_23_B_11.8_Si_10.2_ microwires (*ρ* ≈ 0.45) (**a**) and experimental dependences represented as *σ_a_* (1/*v*^2^) plots (**b**). Adapted from ref. [79].

Considering the magnetoelastic anisotropy contribution evidenced from Figure 8, one can predict an inverse square root *v*(*σ_a_*) dependence. Qualitatively, a decrease in *v* with *σ_a_* for Fe_55_Co_23_B_11.8_Si_10.2_ microwires (*ρ* ≈ 0.45) is observed (see Figure 9a). The experimental results represented as *σ_a_* (*v*^−2^) are shown in Figure 9b. As evidenced from Figure 9b, obtained results cannot be described by single *v*(*σ_a_*^−1/2^) dependence. However, at a sufficiently high *σ_a_*-values observed *v*(*σ_a_*) dependence can be described as 2 *v*(*σ_a_*^−1/2^). One of the possible reasons is that when the *σ_a_* are of the same order as *σ_i_*, the *σ_a_* influence on the DW dynamics cannot be taken into account in such a simple assumption.

**Figure 10:**
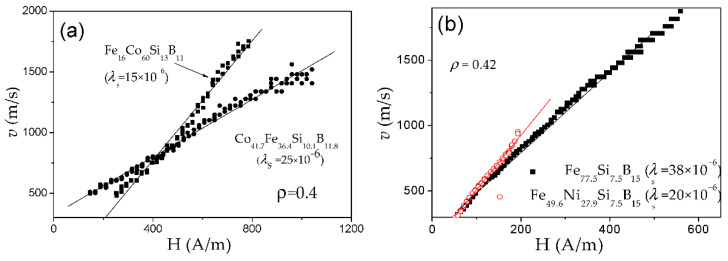
Dependencies of DW velocity, *v*, on magnetic field, H measured in Fe_16_Co_60_Si_13_B_11_ and Co_41.7_Fe_36.4_Si_10.1_B_11.8_ microwires with *ρ* = 0.4 (**a**) and Fe_77.5_Si_7.5_B_15_ and Fe_49.6_Ni_27.9_Si_7.5_B_15_ with *ρ* = 0.42 (**b**). Figure 9a is adapted from ref. [79].

*v*(*H*) dependencies for Fe_16_Co_60_Si_13_B_11_ and Co_41.7_Fe_36.4_Si_10.1_B_11.8_ and Fe_77.5_Si_7.5_B_15_ and Fe_49.6_Ni_27.9_Si_7.5_B_15_ amorphous microwires with the same *ρ*-ratios (0.4 and 0.42) are shown in Figure 10a,b, respectively. For each figure (Figure 10a,b) the difference in *v*(*H*) dependencies must be attributed to different *λ_s_*-values. In both cases, higher *S* is observed for lower *λ_s_* magnitude: for Co_41.7_Fe_36.4_Si_10.1_B_11_ microwire (*λ_s_ ≈* 25 × 10^−6^*) S ≈* 1.2 m^2^/As, while in Fe_16_Co_60_Si_13_B_11_ (*λ_s_ ≈* 15 × 10^−6^) *S ≈* 2.4 m^2^/As (see Figure 10a). The same tendency can be appreciated from Figure 10b: for Fe_77.5_Si_7.5_B_15_ microwire (*λ_s_* ≈ 38 × 10^−6^) *S ≈* 3.06 m^2^/As and in Fe_49.6_N_i27.9_Si_7.5_B_15_ microwire (*λ_s_* ≈ 20 × 10^−6^) *S ≈* 4.53 m^2^/As.

The domain wall mobility from relation (3) is given by

*S* = 2 *μ*_0_*M_s_*/*β* (5)

where *μ*_0_ is the magnetic permeability of vacuum, *M_s_* saturation magnetization, and *β* is the viscous damping coefficient.

The origin of the damping in magnetic microwires and its correlation with *K_me_* is discussed elsewhere [15,16,65]. The micro-eddy currents contribution, *β_e_*, is considered to be negligible for amorphous high resistive materials with thin dimensionality [15,16]. Accordingly, the magnetic relaxation damping, *β_r_*, is considered elsewhere as the main factor affecting the DW dynamics at least in amorphous microwires [15,16,27,28,65]. The magnetic relaxation damping is related to a delayed rotation of electron spins and inversely proportional to the domain wall width and given as [16,27,78]:

*β_r_* ≈ 2*M_s_π*^−1^ (*K_me_*/*A*)^1/2^ (6)

where *A* is the exchange stiffness constant.

Consequently, both, *K_me_* and *M_s_*, can affect *S* magnitude, as we experimentally observed in a few Co-Fe and Fe-Ni -rich microwires. Accordingly, the observed change in *S*-values can be qualitatively explained considering *λ_s_* presented in Figure 10, and the fact that doping with Co and Ni reduces *M_s_* [76].

**Figure 11:**
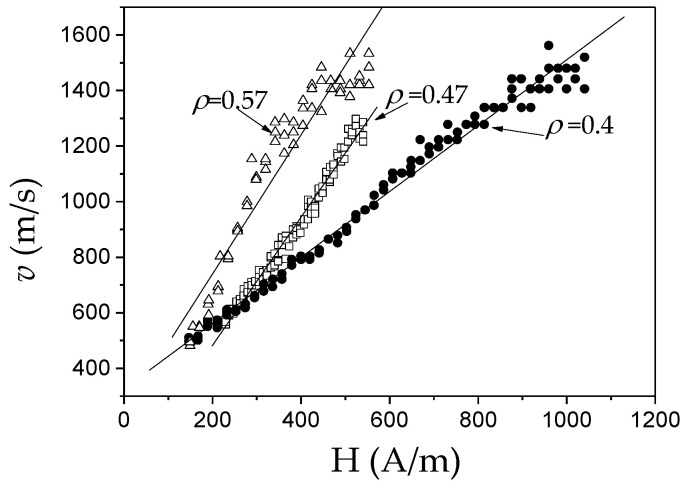
Dependencies of DW velocity, *v*, on the magnetic field, H measured in Fe_55_Co_23_B_11.8_Si_10.1_ microwires with different *ρ*-ratios. Adapted from ref. [21].

The other proof of the important impact of *K_me_* on DW dynamics are experimental *v*(*H*) dependencies measured in microwires of the same chemical composition (i.e., with the same *λ_s_*) but with different *σ_i_* (see Figure 11). As described above, controlling the *ρ*-ratio allows internal stresses to be varied.

Similar to Figure 10, *S* decreases with *σ_i_* increasing (decreasing the *ρ*-ratio), that is, with increasing *K_me_* given by the Equation (4).

**Figure 12:**
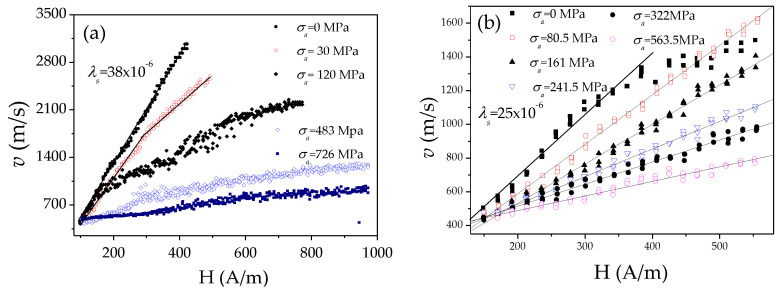

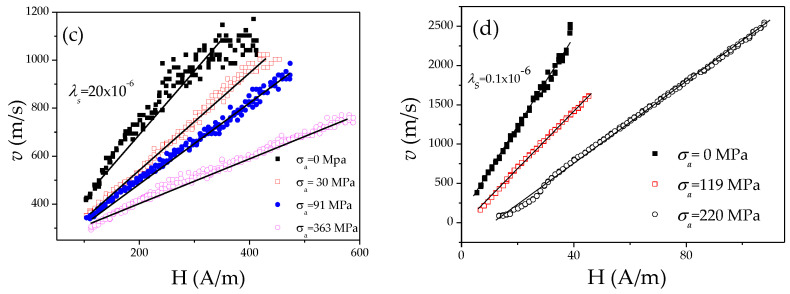
Dependencies of DW velocity, *v*, on magnetic field, H for Fe_74_Si_11_B_13_C_2_ (*ρ* ≈ 0.55) (**a**), Co_41.7_Fe_36.4_Si_10.1_B_11.8_ (*ρ* ≈ 0.57) (**b**) Fe_49.6_Ni_27.9_Si_7.5_B_15_ (*ρ* ≈ 0.42) (**c**) and Co_56_Fe_8_Ni_10_Si_10_B_16_ (*ρ* ≈ 0.42) (**d**) microwires with different *λ_s_* measured under application of applied stresses, *σ*_a_. Reprinted with permission from refs. [28,68,79], respectively.

The robust structure of microwires allows the application of external stresses during annealing or during measurements [80,81]. The application of stresses is the simplest way to modify *K_me_*. Accordingly, the modification in the *v*(*H*) dependences upon stresses, *σ_a_*, the application is another evidence of the *K_me_* impact in the DW dynamics. In Figure 12 are provided *v*(*H*) dependencies measured in microwires with different magnetostriction coefficients, i.e., Fe_74_B_13_Si_11_C_2_ (*λ_s_* ≈ 38 × 10^−6^), Co_41.7_Fe_36.4_Si_10.1_B_11.8_ (*λ_s_* ≈ 25 × 10^−6^), Fe_49.6_Ni_27.9_Si_7.5_B_15_ (*λ_s_* ≈ 20 × 10^−6^) and Co_56_Fe_8_Ni_10_Si_10_B_16_ microwires (*λ_s_* ≈ 0.1 × 10^−6^). A remarkable *v* and *S* decreasing can be observed under *σ_a_* application for all studied microwires.

**Figure 13:**
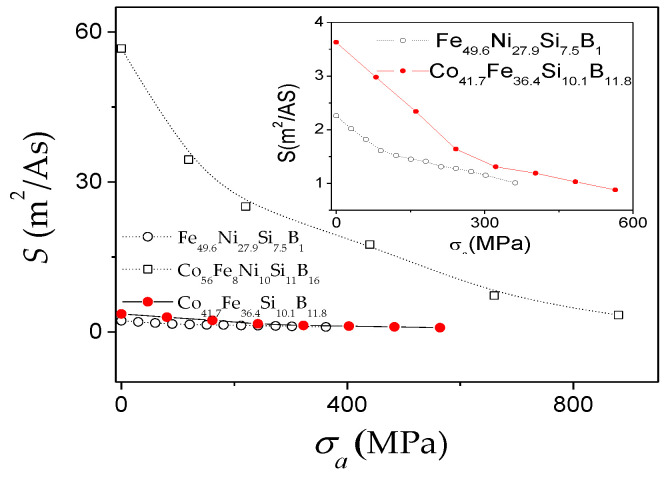
Applied stress, *σ_a_*, the dependence of DW mobility, *S*, evaluated for Co_56_Fe_8_Ni_10_Si_11_B_16_, and Fe_49.6_Ni_27.9_Si_7.5_B_15_microwires. Adapted from ref. [28].

It is worth mentioning, that one of the highest *S* (*S* ≈ 58 m^2^/As) is observed in Co_56_Fe_8_Ni_10_Si_11_B_16_ microwires with low magnetostriction constant (*λ_s_* ≈ 0.1 × 10^−6^): more than an order of magnitude higher *S* is observed in Co_56_Fe_8_Ni_10_Si_11_B_16_ microwire as-compared with Fe_49.6_Ni_27.9_Si_7.5_B_15_ and Co_41.7_Fe_36.4_Si_10.1_B_11.8_ microwires (Figure 13). A considerable decrease in the magnitude of *S* upon applied stresses is observed in all microwires (see Figure 13).

Accordingly, considering experimental results provided in Figure 9, Figure 10, Figure 11, Figure 12 and Figure 13, we can assume that appropriate selection of chemical composition, with low *λ_s_*-value, is one of the effective routes for the DW velocity improvement in magnetic microwires.

An alternative possibility is related to the design of the magnetic anisotropy distribution which is more favorable for the DW dynamics improvement [25,26].

In fact, both, cylindrical geometry and the specific domain structure of magnetic microwires with positive magnetostriction coefficient consisting of a single axially magnetized inner domain surrounded by the outer domain shell with transverse magnetic anisotropy, are the unique conditions for the realization of ultrafast magnetization switching. In magnetic microwires with such domain structure, the magnetization reversal is attributed to the depinning and fast DW propagation within an inner single domain upon application of the external magnetic field.

The noticeably higher *S* observed in the Co_56_Fe_8_Ni_10_Si_10_B_16_ microwires correlates with the lowest *K_me_* due to the low magnetostriction coefficient (*λ_s_* ≈ 0.1 × 10^−6^). However, doping of Fe-rich amorphous alloys by Co or Ni leads to a decrease in the saturation magnetization [76].

**Figure 14:**
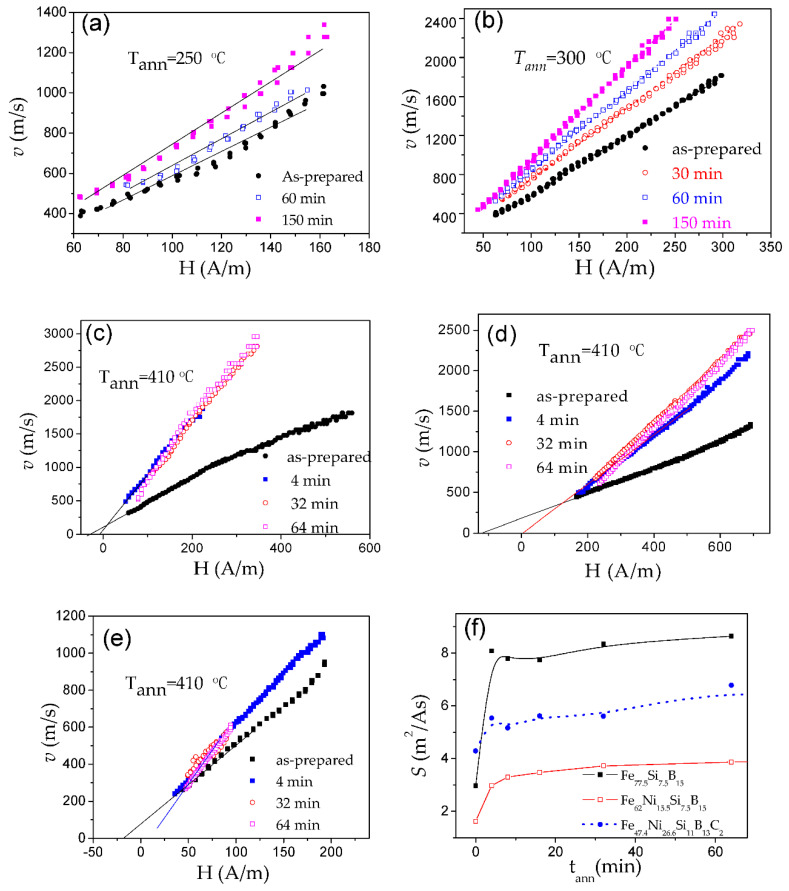
Dependencies of DW velocity, *v*, on magnetic field, H, measured in as-prepared and annealed for different annealing time, *t_ann_*, and annealing temperature, *T_ann_* Fe_74_B_13_Si_11_C_2_ (**a**,**b**) Fe_77.5_Si_7.5_B_15_ (**c**), Fe_62_Ni_15.5_Si_7.5_B_15_ (**d**), Fe_47.4_Ni_26.6_Si_11_B_13_C_2_ (**e**) microwires and *S*(*t_ann_*) for Fe_77.5_Si_7.5_B_15_, Fe_62_Ni_15.5_Si_7.5_B_15_ and Fe_47.4_Ni_26.6_Si_11_B_13_C_2_ microwires annealed at 410 °C (**f**). The lines are just guides for the eyes. Reprinted with permission from refs. [25,82].

Accordingly, various ways have been proposed to optimize the DW dynamics by reducing *K_me_* [25,26,27,28,70,82]. In amorphous materials with a fixed chemical composition, the most common way to reduce *K_me_* is annealing, allowing internal stress relaxation. A remarkable improvement of the DW velocity is observed in various Fe-, Fe-Ni-rich microwires upon annealing [25,70,82]. One of the examples is provided in Figure 7b. The change in the *v*(*H*) dependencies, measured in microwires after annealing under various conditions, is shown in Figure 14a–e.

After annealing, a noticeable increase in the DW velocity and DW mobility was observed in all studied microwires (Figure 14). The most remarkable changes in the *v*(*H*) dependences (i.e., increase in *v* and *S*) are observed in Fe-rich microwires. In addition, the observed changes depend on *t_ann_* and *T_ann_*: faster DW dynamics and more noticeable changes in the *v*(*H*) dependencies are observed with increasing *t_ann_* and *T_ann_* (Figure 14a–f).

Generally, the DW dynamics of Fe-Ni-based alloys are less affected by the annealing. Thus, rather high *S*-values are observed in as-prepared Fe_47.4_Ni_26.6_Si_11_B_13_C_2_ microwire. However, the increase in *S* is more significant upon annealing the Fe_77.5_Si_7.5_B_15_ microwire, and, hence, the annealed Fe_77.5_Si_7.5_B_15_ microwire has higher *S* (see Figure 14f).

**Figure 15:**
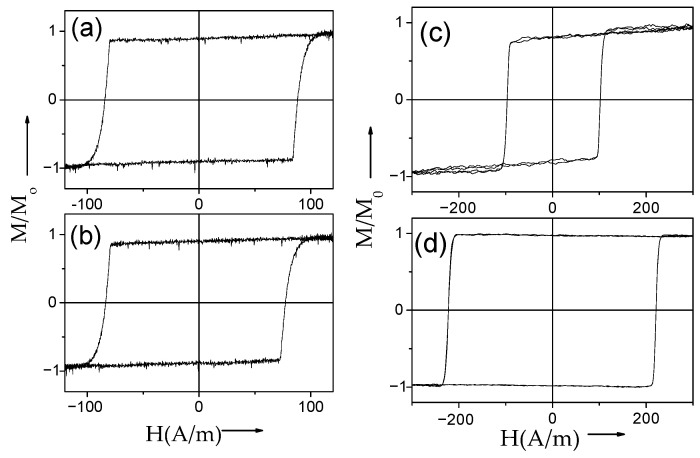
Hysteresis loops of as-prepared (**a**,**c**) and annealed for 180 and 120 min (**b**,**d**) Fe_75_B_9_Si_12_C_4_ and Fe_62_Ni_15.5_Si_7.5_B_15_ microwires, respectively.

For interpretation of the different behavior of Fe-Ni based microwires, several reasons can be considered. As has been reported [52], considerable magnetic hardening is observed in Fe-Ni-based microwires, while slight magnetic softening is reported for Fe-rich microwires annealed at similar conditions. This tendency can be appreciated in Figure 15, where hysteresis loops of as-prepared and annealed Fe_77.5_Si_7.5_B_15_ and Fe_62_Ni_15.5_Si_7.5_B_15_ microwires are shown.

One of the reasons for the different behavior of annealed Fe_77.5_Si_7.5_B_15_ and Fe_62_Ni_15.5_Si_7.5_B_15_ microwires is the local nano-sized precipitations reported for annealed Fe-Ni based microwires [83].

Additionally, as discussed elsewhere [76], the annealing influence is not limited to stresses relaxation. Annealing at a temperature below the Curie temperature, *T_c_*, can produce induced anisotropies along the direction of local spontaneous magnetization inside ferromagnetic domains [76]. Such induced anisotropies produced by annealing are stronger in amorphous alloys containing at least two transition metals than for those with only one transition metal [76,87]. In particular, magnetic hardening of Fe-Ni, Fe-Co or Co-Fe-Ni based amorphous alloys associated with the DWs stabilization has been observed after annealing at temperatures below the *T_c_* [76,87]. There are several mechanisms of such DW stabilization related to composition re-organization, like atom pair ordering in Fe-Ni and Fe-Co based amorphous materials, metalloids diffusion, directional compositional short-range atomic ordering, or topological atomic ordering [84,85,86,87].

Additionally, Fe-Ni amorphous alloys have lower *λ_s_* and *M*_s_. Accordingly, *β_r_* given by Equation (6) is less affected by stress relaxation.

Several publications report that the DW velocity of various kinds of magnetic wires can be improved by applying a transversal magnetic field [88,89,90]. Such influence is attributed to the effect of the transverse magnetic field on the spin precession and DW width [88,89].

**Figure 16:**
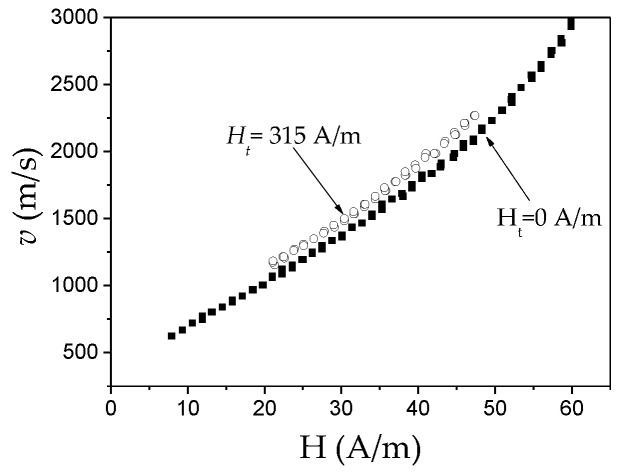
The magnetic field, H, the dependence of DW velocity, *v*, in Co_56_Fe_8_Ni_10_Si_11_B_16_ microwire measured without transverse magnetic field and under an applied transverse magnetic field, *H_t_*. Adapted from ref. [90].

Some increase in the DW velocity for the Co_56_Fe_8_Ni_10_Si_11_B_16_ microwire upon a transverse magnetic field, *H_t_*, is reported [90], however, the DW mobility does not change significantly in this case (see Figure 16).

**Figure 17:**
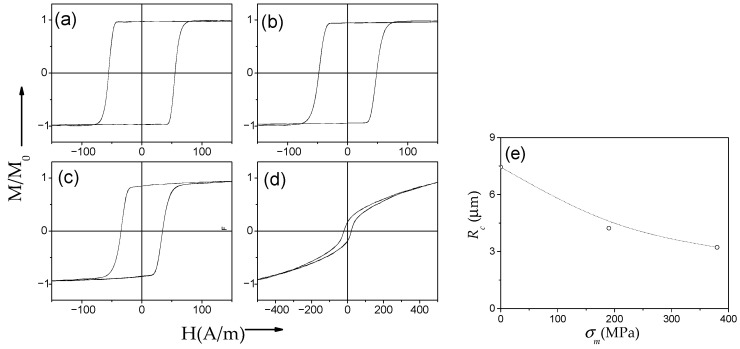
Hysteresis loops of as-prepared (**a**), annealed at *T_ann_* = 300 °C for *σ_m_*= 0 MPa (**b**), stress-annealed at *T_ann_* = 300 °C for *σ*_m_ =190 MPa (**c**) and at *T_ann_* = 300 °C for *σ_m_* = 380 MPa Fe_75_B_9_Si_12_C_4_ sample (**d**) and inner axially magnetized core radius, *R_c_*, dependence on stress, *σ_m_*, applied during the annealing (**e**). Reprinted with permission from ref. [28].

On the other hand, the considerable impact of transverse magnetic anisotropy induced by stress-annealing on hysteresis loops and DW dynamics is reported [27,28,52,58,91]. Stress-annealing is a quite efficient method for tuning of hysteresis loops of Fe-rich microwires: remarkable change of not only the coercivity but even of the character of hysteresis loops of Fe_75_B_9_Si_12_C_4_ microwire after stress annealing is shown in Figure 17.

Thus, rectangular hysteresis loops observed in as-prepared and annealed (without stress) Fe_75_B_9_Si_12_C_4_ microwires transform into inclined hysteresis loops with a rather low coercivity after stress annealing (see Figure 17d). Such transformation is observed either at high enough *T_ann_*, *t_ann_*, or *σ_m_* [27,28,81]. However, the hysteresis loop of microwires annealed at a moderate stress applied during annealing is still rectangular with lower *H_c_* (see Figure 17c).

Stress-annealed Fe_75_B_9_Si_12_C_4_ microwires with a rectangular hysteresis loop present lower *H_c_* and squareness ratio, *M_r_*/*M*_0_, as compared to as-prepared and even annealed at the same conditions (*T_ann_* and *t_ann_*) microwires (see Figure 17).

Commonly, the domain structure of magnetic wires is described in terms of the core-shell model as consisting of an inner axially magnetized core and an outer domain shell with radial magnetization orientation [17,90,92,93,94]. The inner axially magnetized core radius, *R_c_*, is related to *M_r_*/*M*_0_ as [17,93]:

*R_c_* = *R*(*M_r_*/*M*_0_)^1/2^, (7)

where *R* is the microwire radius.

Consequently, *R_c_*(*σ_m_*) dependence evaluated from Figure 17a–d using Equation (7) is also shown in Figure 17e. From the evaluated *R_c_*(*σ_m_*) dependence, we can assume an increase in the volume of the microwire with transverse magnetic anisotropy at expense of the inner axially magnetized core with the increase in *σ_m_*. Such modification of the spatial distribution of magnetic anisotropy is also evidenced by the remarkable improvement of the GMI ratio, Δ*Z*/*Z*, and modification of magnetic field dependence of Δ*Z*/*Z* in stress-annealed Fe_75_B_9_Si_12_C_4_ microwires [27,81,95].

**Figure 18:**
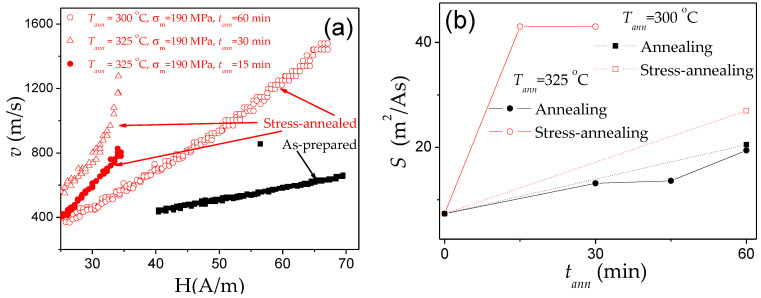
Dependencies of DW velocity, *v*, on the magnetic field, H, measured in as-prepared and stress-annealed at *T_ann_* = 300 °C and 325 °C for *σ_m_* = 190 MPa and different *t_ann_* Fe_75_B_9_Si_12_C_4_ microwires (**a**) and evolution of DW mobility, *S*, upon stress-annealing and conventional annealing of Fe_75_B_9_Si_12_C_4_ microwires (**b**). Adapted from ref. [27].

As can be appreciated from Figure 18a, stress-annealing allows remarkable improvement of the DW velocity. Comparison of *S* obtained in annealed and stress-annealed Fe_75_B_9_Si_12_C_4_ microwires is provided in Figure 18b. As can be appreciated, stress-annealing allows an increase in *S*-values up to 47 m^2^/As (see Figure 18b).

Observed remarkable improvement of the DW dynamics (*S* and *v*-values) has been attributed to the transverse magnetic anisotropy of the outer domain shell that similarly affects the traveling DW as the application of transversal bias magnetic field that allows the DW velocity enhancement [26,35].

Accordingly, the DW dynamics of magnetic microwires with positive magnetostriction coefficient exhibiting spontaneous magnetic bistability can be considerably improved either by minimization of the magnetoelastic anisotropy, transverse magnetic field or by annealing, allowing internal stresses relaxation. Further DW dynamics improvement can be achieved by stress-annealing, allowing induction of transverse magnetic anisotropy.

#### 3.1.2. DW Propagation in Amorphous Microwires with Annealing-Induced Magnetic Bistability

- Magnetic field driven DW propagation in Co-rich amorphous microwires
- Current driven DW propagation in Co-rich amorphous microwires

**Figure 19:**
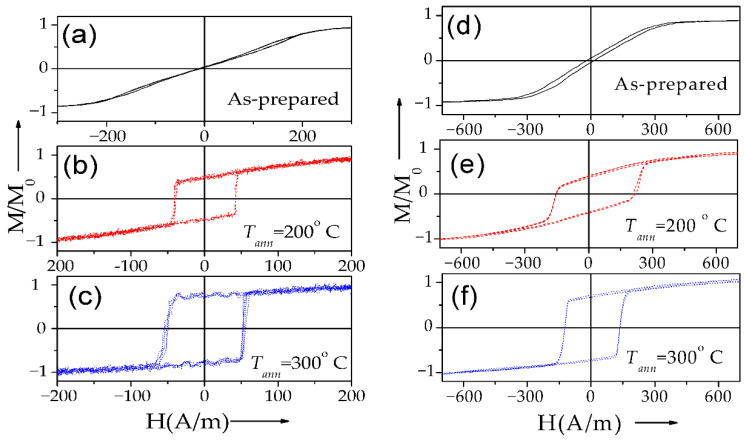
Hysteresis loops of as-prepared (**a**,**d**) and annealed for 5 min at different temperatures Co_69.2_Fe_4.1_B_11.8_Si_13.8_C_1.1_ (**b**,**c**) and Fe_8.1_Co_50.7_Ni_17.6_B_13.3_Si_10.3_ (**e**,**f**) microwires. Reproduced with permission from ref. [28].

DW propagation in magnetic microwires with magnetic bistability induced by annealing is relatively a recent topic: the transformation of linear hysteresis loop into rectangular is reported in various Co-rich microwires with vanishing and negative *λ_s_* [26,28,96,97]. Indeed, perfectly rectangular hysteresis loops have been observed in various Co-rich microwires after appropriate annealing (see Figure 19). Such a remarkable change of hysteresis loops of Co-rich microwires is discussed considering the effect of internal stress relaxation on *λ_s_* value and sign [26,28,96,97,98]. Accordingly, a single DW propagation is observed in such Co-rich microwires with annealing-induced magnetic bistability [6,26,28].

**Figure 20:**
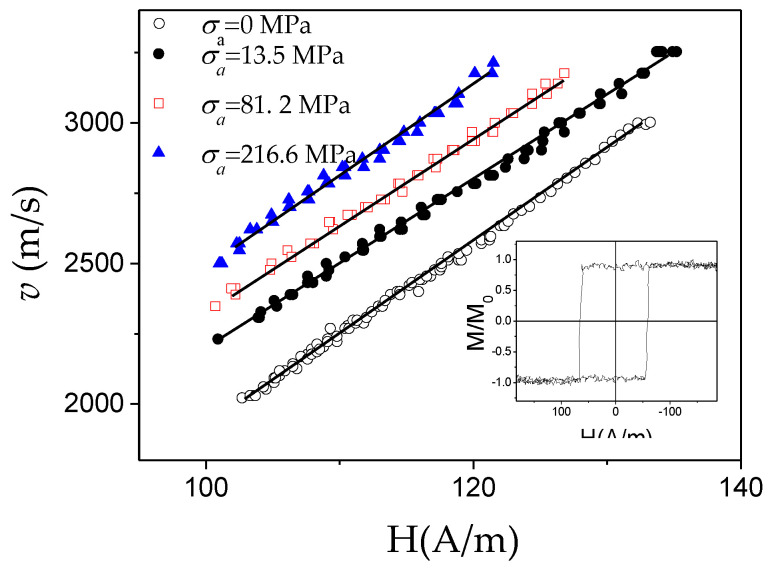
Dependencies of DW velocity, *v*, on the magnetic field, H, measured in Co_69.2_Fe_4.1_B_11.8_Si_13.8_C_1.1_ microwires annealed at *T_ann_* = 300 °C for 45 min measured under different applied stresses. Adapted from ref. [28].

A specific feature of the DW dynamics in Co-rich microwires with annealing-induced magnetic bistability is the character of the stress dependence of the DW dynamics. A considerable increase in the DW velocity upon applied tensile stress has been reported for various Co-rich microwires with annealing-induced magnetic bistability [28]. Such unusual stress influence on *v*(*H*) dependencies of Co_69.2_Fe_4.1_B_11.8_Si_13.8_C_1.1_ glass-coated microwires (*ρ* = 0.85) annealed at *T_ann_* = 300 °C (*t_ann_* = 45 min) is shown in Figure 20.

Such stress dependence of DW dynamics is opposite to that reported for magnetic microwires with positive *λ_s_* (see Figure 12). Additionally, high *v*-values (up to 3.5 km/s) can be observed in such Co-rich microwires.

**Figure 21:**
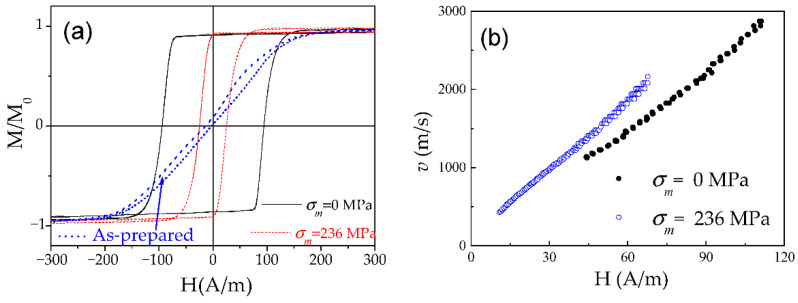
Hysteresis loops (**a**) and dependencies of DW velocity, *v*, on magnetic field, H, (**b**) of Co_69.2_Fe_3.6_Ni_1_B_12.5_Si_11_Mo_1.5_C_1.2_ (d = 22.8 μm, D = 23.2 μm) annealed and stress-annealed at *T_ann_* = 350 °C for 1 h. Reprinted with permission from ref. [28].

As in the case of Fe-rich microwires, stress annealing of Co-rich microwires significantly affects the hysteresis loops [28,97]. Typically, Co-rich stress-annealed microwires have lower *H_c_* than the same microwires annealed at the same *T_ann_* (see Figure 21a for Co_69.2_Fe_3.6_Ni_1_B_12.5_Si_11_Mo_1.5_C_1.2_ sample).

**Figure 22:**
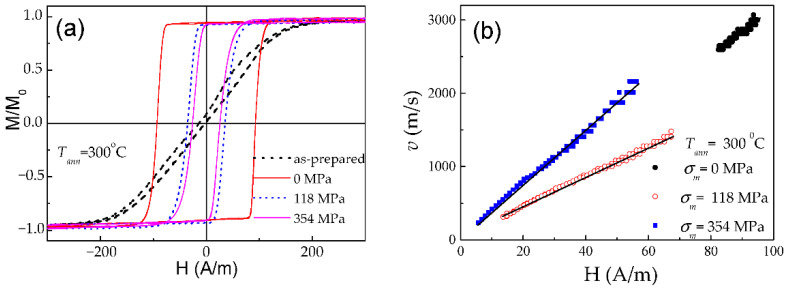
Hysteresis loops (**a**) dependencies of DW velocity, *v*, on magnetic field, H, (**b**) measured for the Co_69.2_ Fe_3.6_Ni_1_B_12.5_Si_11_Mo_1.5_C_1.2_ samples annealed at *T_ann_* = 300 °C for *σ_m_* = 0 MPa, *σ_m_* = 118 MPa and *σ_m_* = 354 MPa. Reprinted with permission from ref. [28].

Accordingly, stress-annealing affects the *v*-value and the linear *v*(*H*) dependence extension (see Figure 21b). The shift of linear *v*(*H*) dependence in the low field region, observed for some stress-annealed Co-rich microwires (see Figure 21b), must be attributed to lower coercivity (see Figure 21a and Figure 22a) as well as to the effect of stress annealing on *H_n_*-value. Considerable increase in *S* in Co-rich microwires with annealing-induced anisotropy up to *S* ≈ 35.5 m^2^/As in stress-annealed (at *σ_m_* = 354 MPa) can be appreciated from Figure 22b. For different stress-annealing conditions, *S* ≈ 40 m^2^/A∙s have been reported in the same microwire [58].

**Figure 23:**
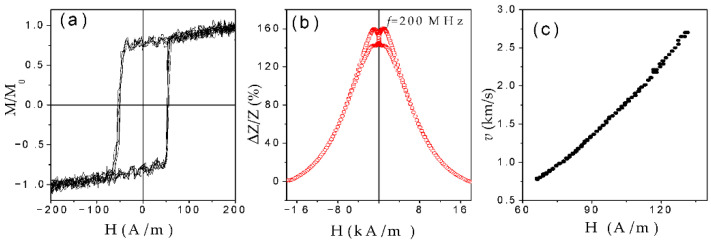
Hysteresis loop (**a**) magnetic field dependence of the GMI ratio measured at 200 MHz (**b**) and dependencies of DW velocity, *v*, on the magnetic field, H, (**c**) measured in annealed at 300 °C (for 5 min.) Co_69.2_Fe_4.1_B_11.8_Si_13.8_C_1.1_ microwire. Adapted from ref. [99].

One more peculiarity of Co-rich microwires with annealing-induced magnetic bistability is that such Co-rich microwires still present quite a high GMI effect, despite the rectangular character of hysteresis loops (see Figure 23a,b) [28,96,97,98,99]. However, single DW propagation upon application of an axial magnetic field can be observed in the same microwire (see Figure 23c). Such unusual combination of magnetic properties is observed in several annealed and stress-annealed Co-rich microwires, i.e., in annealed and stress-annealed Co_69.2_Fe_4.1_B_11.8_Si_13.8_C_1.1_ (see Figure 23), Co_50_*_._*_69_Fe_8_*_._*_13_Ni_17_*_._*_55_B_13_*_._*_29_Si_10_*_._*_34_ [97] or Co_69.2_Fe_3.6_Ni_1_B_12.5_Si_11_Mo_1.5_C_1.2_ microwires [58,98].

**Figure 24:**
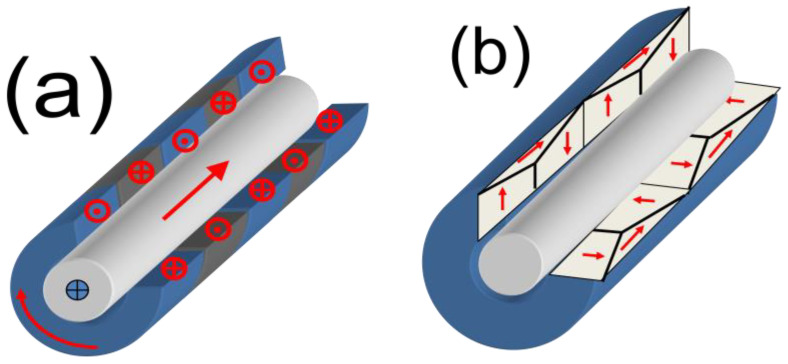
Schematic domain structure of (**a**) annealed Co-rich microwire and (**b**) Fe-rich microwire. Adapted from ref. [17].

Such difference with Fe-rich microwires is explained considering different domain structures of Co-rich microwires with annealing-induced magnetic bistability and Fe-rich microwires with spontaneous magnetic bistability [17]. The common feature of both kinds of microwires is the existence of the inner axially magnetized core responsible for single DW propagation (see Figure 24). However, the magnetization in the outer domain shell is considered different: radial for Fe-rich microwires and circumferential for Co-rich microwires with annealing-induced magnetic bistability [17].

The GMI hysteresis reported in Co-rich microwires (also visible in Figure 23b) has been explained by the magnetostatic interaction of the inner axially magnetized core and the outer domain shell with circumferential magnetization orientation. Accordingly, we assumed that the AC electrical current producing circumferential AC magnetic field must affect the magnetization of the outer shell with circumferential magnetization [17]. In the annealed (*T_ann_* = 300 °C for *t_ann_* = 5 min) Co_69_Fe_4_B_12_Si_14_C_1_ microwire (d = 25 µm) the current with an amplitude of 10.5 mA produces AC circumferential magnetic field, *H_circ_*, given by the formula:

*H_circ_* = *I*/2*πr* (8)

where *I* is the current value and *r* the radial distance.

The estimated AC magnetic field in the surface of the metallic nucleus was *H_circ_* ≈ 134 A/m. Accordingly, instead of a magnetizing coil, we used an AC flowing through the microwire and tried to evaluate the DW propagation in this microwire using 3 pick-up coils.

**Figure 25:**
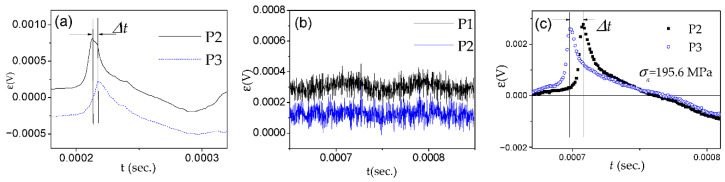
Voltage peaks induced by the magnetization change in the pick-up coils in annealed Co_69_Fe_4_B_12_Si_14_C_1_ (**a**) and as-prepared Fe_75_B_9_Si_12_C_4_ (**b**) microwires and in annealed Co_69_Fe_4_B_12_Si_14_C_1_ microwire (**c**) under *σ_a_* ≈ 195.6 MPa. Adapted from ref. [17].

As can be observed in Figure 25a–c, appreciable electro-motive force (EMF) peaks, ε, generated in the pick-up coils by the magnetization changes caused by the current in annealed Co_69_Fe_4_B_12_Si_14_C_1_ sample are observed. However, in the as-prepared Fe_75_B_9_Si_12_C_4_ microwire, the EMF voltages induced in all pick-up coils are quite small.

For interpretation of the observed dependences, we considered different domain structure of annealed Co-rich and as-prepared Fe-rich microwires (see Figure 24). In fact, both samples present similar bulk hysteresis loops [17].

The negligible EMF signals observed in Fe-rich microwires can be explained by considering that the circular magnetic field generated by the electric current does not sufficiently affect the radially magnetized outer domain of Fe_75_B_9_Si_12_C_4_ microwires.

In contrast, the appreciable EMF peaks induced in the pick-up coils have been attributed to the magnetization change in the outer domain shell of annealed Co_69_Fe_4_B_12_Si_14_C_1_ microwire induced by *H_circ_* produced by electrical current (Oersted field).

The systematic temporal shift, Δ*t*, between the EMF peaks for the annealed Co_69_Fe_4_B_12_Si_14_C_1_ sample (Figure 25a) becomes even more evident under the effect of applied tensile stress in the annealed Co_69_Fe_4_B_12_Si_14_C_1_ sample (Figure 25c).

**Figure 26:**
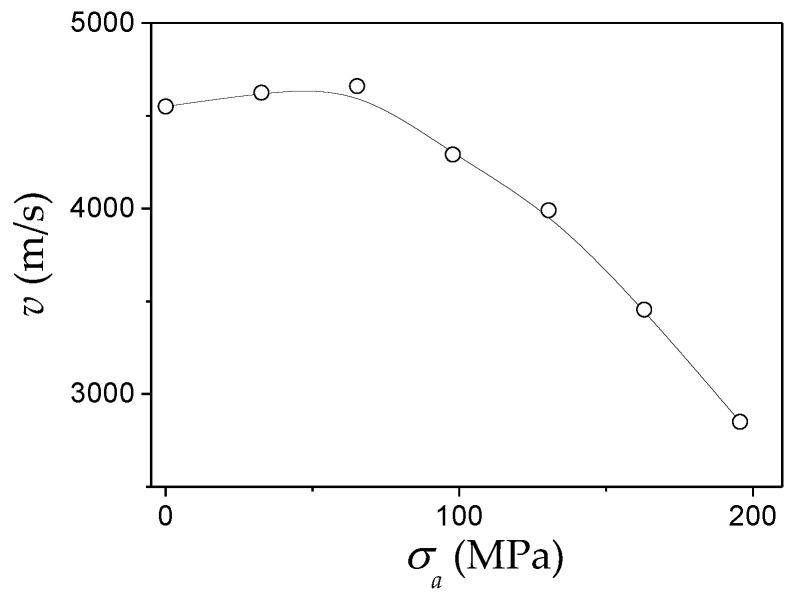
Dependence of DW velocity, *v*, on applied tensile stress, *σ_a_*, estimated for annealed Co_69_Fe_4_B_12_Si_14_C_1_ microwire. Reprinted from ref. [17].

Moreover, from the shift between the peaks, the velocity of the DW velocity from Equation (3) and its stress dependence have been evaluated (see Figure 26). Quite high *v* (about 4.5 km/s) and its non-monotonic stress dependence are observed.

Accordingly, the origin of current-induced DW propagation in Co-rich microwires with annealing-induced magnetic bistability is quite different from that reported in planar nanowires [9,10] and must be attributed to magnetostatic interaction between the outer circumferentially magnetized domain shell an inner axially magnetized core [17,100,101].

As can be appreciated from the above presented experimental results and discussion on DW dynamics in microwires with spontaneous magnetic bistability and positive magnetostriction coefficient, DW dynamics can be effectively tuned either by careful selection of microwire composition or annealing conditions. Annealing and stress-annealing allow further DW dynamics optimization in magnetic microwires with spontaneous magnetic bistability.

#### 3.1.3. Single DW Propagation in Nanocrystalline Microwires

- DW propagation in Finemet-type amorphous and nanocrystalline microwires
- DW propagation in as-prepared nanocrystalline microwires
- Impact of saturation magnetization on DW mobility in nanocrystalline microwires

Although in most cases, significant degradation of the magnetic softness of amorphous precursor upon the crystallization is observed, the formation of nano-sized crystallites with an average grain size of 10–15 nm in the amorphous matrix is observed in Fe–(Si,B) alloys doped by Cu and Nb [76,102]. Accordingly, the term “nanocrystalline alloys” is presently used for the materials with a majority of average grain sizes between 1 and 50 nm [103]. Such nanocrystalline materials can be prepared by several methods including rapid solidification, devitrification of amorphous materials by thermal treatment or deposition techniques [76,102,103].

As in the case of amorphous microwires, the magnetocrystalline anisotropy in nanocrystalline microwires is negligible, because it is averaged out, since the intrinsic exchange length (about 35 nm) is larger than the average grain size (usually about 10 nm). Accordingly, the magnetoelastic and shape anisotropies of nanocrystalline microwires are dominant [76].

The most common nanocrystalline materials are FeSiBCuNb alloys, known as Finemet [76,102], FeB-M-Cu (M = Zr, Nb, Hf) alloys called Nanoperm, and, more recently, FeCoB-M-Cu alloys, called Hitperm [103,104].

The advantages of nanocrystalline alloys are high saturation magnetization, vanishing magnetic anisotropy (K) and in some cases low *λ_s_* [76,102,103,104].

**Figure 27:**
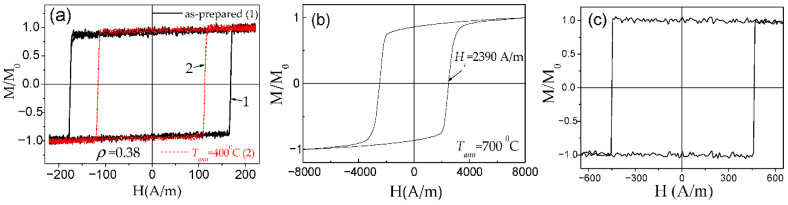
Hysteresis loops of as-prepared and annealed at 400 °C Fe_70.8_Cu_1_Nb_3.1_Si_14.5_B_10.6_ (*ρ* = 0.38) (**a**) annealed at 700 °C Fe_71.8_Cu_1_Nb_3.1_Si_15_B_9.1_ (*ρ* = 0.36) (**b**) and Fe_38.5_Co_38.5_B_18_Mo_4_Cu_1_ (*ρ* = 0.6) (**c**) microwires. Adapted from refs [41,111,114], respectively.

Better magnetic softness and even high GMI effect are reported in Finemet-type microwires [105,106,107]. Additionally, as-prepared and even devitrified Finemet-type and Hitperm-type microwires can exhibit perfectly rectangular hysteresis loops (see Figure 27) [41,108,109,110,111,112,113,114].

Accordingly, single DW propagation has been observed in various nanocrystalline microwires.

The main interest in Finemet alloys is the combination of high saturation magnetization with vanishing *λ_s_*. The latter is commonly attributed to the coexistence of residual amorphous phase with positive magnetostriction, $\lambda_{s}^{am}$, ($\lambda_{s}^{am}\approx$ 20 × 10^−6^) and α-Fe-Si nanocrystals with negative magnetostriction, $\lambda_{s}^{FeSi}$, ($\lambda_{s}^{FeSi}\approx$ −6 × 10^−6^) [115] giving rise to vanishing net magnetostriction values, according to [76,115]:

(9) $$\lambda_{s}^{eff}\;\approx \;V_{cr}\;\lambda_{s}^{FeSi}+\;\left(1-\;V_{cr}\right)\;\lambda_{s}^{am}$$

where *λ_s_^eff^* is the net magnetostriction coefficient, and *V_cr_* the crystalline volume fraction.

In fact, $\lambda_{s}^{FeSi}$ depends on the Si-content in the nanocrystalline state. Therefore, *λ_s_^eff^* is also affected by the composition of the nanocrystalline phase.

Consequently, nanocrystallization allows a decrease in the magnetoelastic anisotropy, *K_me_*, given by Equation (4).

The relevant parameter allowing magnetic softening of nanocrystalline materials is the correlation between the average inter-grain distance, *d_i_*, and the exchange length, *L_ex_*, of the precipitating α-FeSi phase. When *d_i_* ≤ *L_ex_*, the α-FeSi grains are exchange-coupled and exhibit collective magnetic behavior. Therefore, several parameters, such as the average grain size, chemical composition, and spatial distribution are relevant for achieving magnetic softening of Finemet-type alloys [50,109,116].

**Figure 28:**
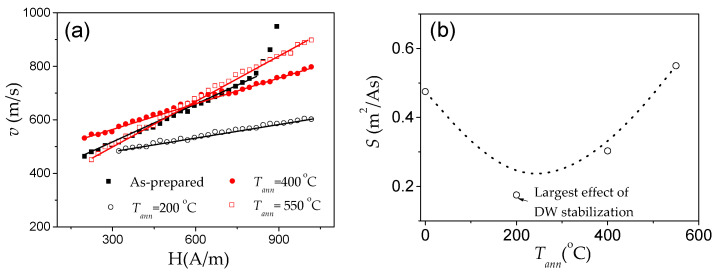
Dependence of DW velocity, *v*, on the magnetic field, *H*, of as-prepared and annealed at different *T_ann_* Fe_73.5_Cu_1_Nb_3_Si_11.5_B_11_ microwires measured at 0 °C (**a**) and dependence of DW mobility, *S*, on annealing temperature, *T_ann_*, evaluated for the same microwires (**b**). Adapted from refs. [50,109].

As can be evaluated from Figure 28, devitrification (achieved by annealing at 550 °C) allows an increase in maximum DW velocity in the linear *v*(*H*) dependence regime (in which the single DW propagation is ensured) from 700 m/s to almost 1000 m/s [50,109]. Such an increase in the maximum DW velocity is associated with an expansion of the linear *v*(*H*) dependence region. The *S*-value evaluated for the linear segment in *v*(*H*) dependencies exhibits non-monotonic dependence on *T_ann_*. *S* ≈ 0.48 m^2^/A∙s is observed in as-prepared Fe_73.5_Cu_1_Nb_3_Si_11.5_B_11_ microwires. Lowest *S* approximately 0.2 m^2^/A∙s is observed in amorphous Fe_73.5_Cu_1_Nb_3_Si_11.5_B_11_ microwire (*ρ* ≈ 0.36) annealed at *T_ann_* = 200 °C, with *S* increasing up to 0.5 m^2^/A∙s for the linear segment in *v*(*H*) dependence in the nanocrystalline microwire (*T_ann_* = 550 °C) [50,109]. The influence of annealing below the Curie temperature, *T_c_* (*T_c_* ≈ 330 °C), has been explained by considering two effects [109]. First, DWs can be stabilized via locally induced magnetic anisotropy (although DW stabilization is commonly considered in the context of amorphous alloys with two or more ferromagnetic elements [84,85]). Therefore, the decrease in *S*-values upon annealing at *T_ann_* = 200 °C can be associated with DW stabilization in Fe_73.5_Cu_1_Nb_3_Si_11.5_B_11_ microwire annealed at *T_ann_* < *T_c_*. In addition, when the sample is cooled to room temperature, internal stresses arise due to the different coefficients of thermal expansion of the glass coating and the metallic nucleus. The domain structure disappears above *T_c_* hence, when annealing at *T_ann_* = 400 °C, local defects will be randomly distributed destabilizing the domain structure. Additionally, this phenomenon leads to relief of the internal stress introduced during microwires production and homogenization of the structure, therefore a large increase in the value of mobility is found [109]. Accordingly, the increase in *v* and *S* upon annealing at *T_ann_* > *T_c_* has been associated with stress relaxation and DW destabilization [109]. The *v*(*H*) dependence in nanocrystalline Fe_73.5_Cu_1_Nb_3_Si_11.5_B_11_ microwire (annealed at 550 °C) is less affected by measuring temperature, an effect that can be attributed to vanishing *λ_s_* [109].

Similarly, faster DW propagation is reported in ultrathin Finemet-type microwires annealed at 550 °C. However, the largest values of *v* do not correlate with the most effective annealing (i.e., at *T_ann_* = 550 °C the grains remain decoupled since *d_i_* > *L_ex_*) [116]. Accordingly, as in the case of amorphous Fe-rich microwires, the contribution from the internal stress relaxation can play an important role in optimizing DW velocity in the case of Finemet-type micrometric and submicrometric wires.

The main problem that restricts the application possibilities of Finemet-type alloys prepared by nanocrystalization of the amorphous precursor is that they are extremely brittle [41]. Poor mechanical properties of nanocrystalline Finemet-type microwires can be avoided by the preparation of nanocrystalline microwires directly by rapid melt quenching. Such nanocrystalline or metastable microwires can be prepared if the quenching rate achieved during the rapid quenching process is not sufficiently high for the preparation of amorphous microwires [31]. Additionally, *S*, given by Equation (5), can be further improved if higher *M_s_* can be achieved.

Higher *M_s_* in Fe-based alloys can be obtained by Co doping [117]. Additionally, highly *M_s_* nanocrystalline Fe_83.7_Si_4_B_8_P_3.6_Cu_0.7_ alloy has been developed [118]. Accordingly, magnetic properties and DW dynamics of new (Fe_0.7_Co_0.3_)_83.7_Si_4_B_8_P_3.6_Cu_0.7_ microwire with nanocrystalline structure (average grain size about 38 nm) have been recently reported [49].

**Figure 29:**
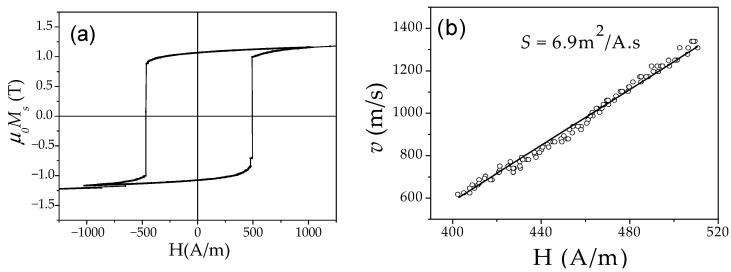
Hysteresis loop (**a**) and dependence of DW velocity, *v*, on the magnetic field, H, (**b**) of the (Fe_0.7_Co_0.3_)_83.7_Si_4_B_8_P_3.6_Cu_0.7_ microwire. Reprinted with permission from ref. [49].

The hysteresis loops of as-prepared (Fe_0.7_Co_0.3_)_83.7_Si_4_B_8_P_3.6_Cu_0.7_ microwire with nanocrystalline structure is presented in Figure 29a.

In spite of the elevated *H_c_*-values, the (Fe_0.7_Co_0.3_)_83.7_Si_4_B_8_P_3.6_Cu_0.7_ microwires present perfectly rectangular hysteresis loops and hence, single DW propagation is evidenced by linear *v*(*H*) dependence (see Figure 29b). As compared to Finemet microwires, (Fe_0.7_Co_0.3_)_83.7_Si_4_B_8_P_3.6_Cu_0.7_ microwire presents higher DW velocity (up to 1400 m/s) and rather high *S ≈* 6.9 m^2^/As. The obtained *S*-value is almost an order of magnitude higher than that obtained in the linear segment of *v*(*H*) dependence for Finemet-type microwire and twice higher than reported in as-prepared Fe-rich amorphous microwire (see Figure 14f).

The reason for this unusually high *S* can be understood considering enhanced saturation magnetization value and its relationship with DW mobility given by Equation (5).

The other family of nanocrystalline materials is Hitperm alloys with chemical composition Fe-Co-*X*-B-(Cu) where *X* is Nb, Zr, Hf, Mo [103]. Hitperm alloys are characterized by high *T_c_* due to Co addition (over 1100 °C) and therefore are suitable for high-temperature applications [103].

**Figure 30:**
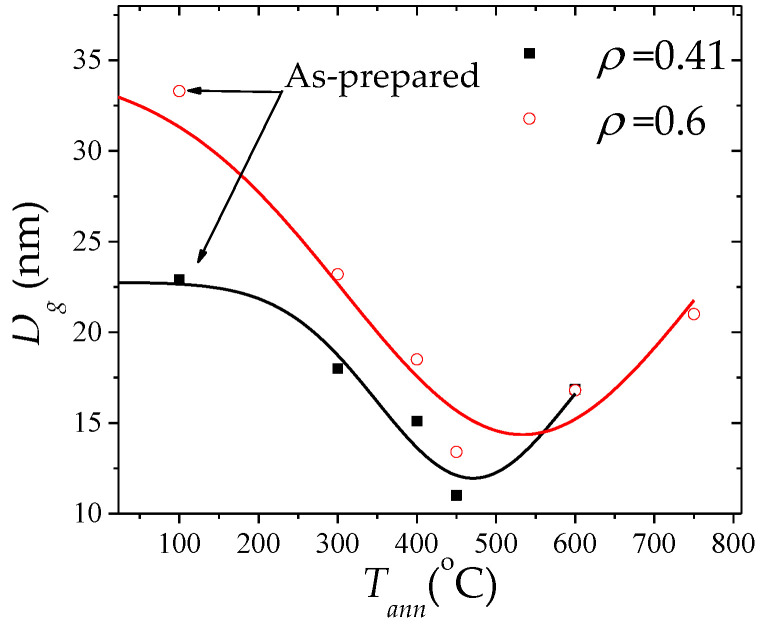
Dependence of average grain size, *D_g_*, on *T_ann_* of Fe_38.5_Co_38.5_B_18_Mo_4_Cu_1_ microwires. Adapted from ref. [122].

Magnetic properties of several Hitperm-type microwires have been reported [119,120,121]. Considering the significant influence of the Cu doping on the formation of nanocrystalline materials, Fe_38.5_Co_38.5_B_18_Mo_4_Cu_1_ microwires with a nanocrystalline structure consisting of α-FeCo phase (average grains size, *D_g_*, 23–33 nm evaluated from the XRD patterns using the Debye-Scherrer formula) embedded in the amorphous matrix have been obtained directly after casting [122,123]. The peculiarity of such microwires is that the annealing allowed further grains size refinement (*D_g_* up to 11 nm, see Figure 30).

Observed *D_g_* decreasing upon annealing has been discussed considering either multiple nucleations of small grains or unstable structures of nanocrystals obtained during microwire preparation [122,123].

**Figure 31:**
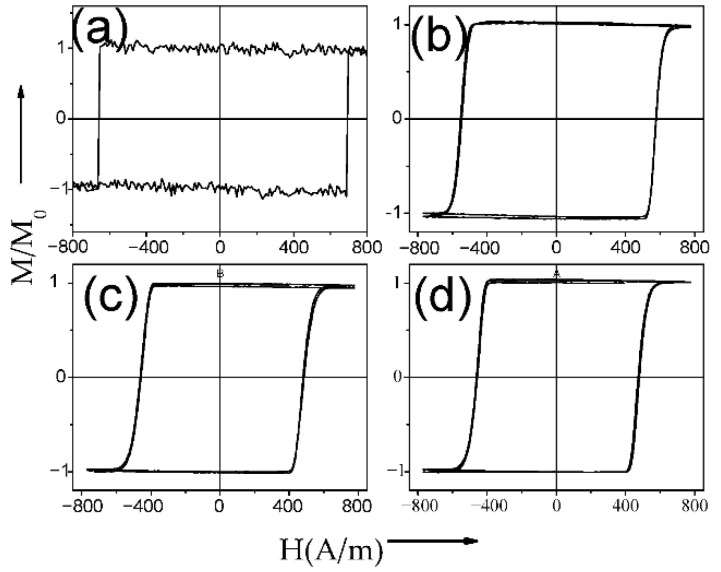
Hysteresis loops of as-prepared (**a**) and annealed at *T_ann_* = 300 °C (**b**), 450 °C (**c**), and 500 °C (**d**) Fe_38.5_Co_38.5_B_18_Mo_4_Cu_1_ microwires (*ρ* = 0.6). Adpated from ref [31].

**Figure 32:**
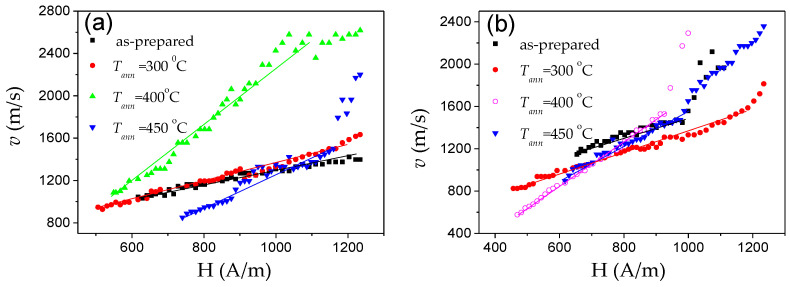
Dependence of DW velocity, *v*, on magnetic field, H, of as prepared and annealed Fe_38.5_Co_38.5_B_18_Mo_4_Cu_1_ microwires *ρ* = 0.41 (**a**) and *ρ* = 0.6 (**b**). Reprinted with permission from ref. [123].

Such Fe_38.5_Co_38.5_B_18_Mo_4_Cu_1_ microwires show perfectly rectangular hysteresis loops in as-prepared and annealed microwires (see Figure 31) and hence single DW propagation (see Figure 32). Observed *D_g_*(*T_ann_*) dependence together with positive magnetostriction coefficient of both the amorphous matrix and the α-FeCo nanograins can explain the considerable magnetic softening observed upon annealing (see Figure 31).

The DW velocity enhancement is observed after annealing (see Figure 32).

An enhancement of the DW velocity as well as mobility after annealing has been attributed to the stress relaxation and grains refinement.

Accordingly, properly prepared and annealed nanocrystalline microwires can exhibit rather fast single DW propagation.

#### 3.1.4. Manipulation of DW Dynamics in Magnetic Microwires

- Evaluation of DW shape and its correlation with microwire properties
- Manipulation of DW shape by control of magnetic anisotropy
- Engineering of DW propagation by local stress-annealing
- DW collision

Understanding of extremely high DW velocities reported for magnetic microwires requires knowledge on the propagating DW structure: shape, width, and on most favorable magnetic anisotropy for the realization of such fast DW propagation. Additionally, an array of metallic rings placed on the microwire can be suitable to encode information [124]. However, the DW must be sufficiently abrupt, because the DW width may limit the information density that can be read successfully. Therefore, the DW width and structure are critical for magnetic tag applications [124].

Accordingly, several attempts to evaluate the features (DW shape, width, etc.) have been performed.

The spatial structure of a propagating DW has been analyzed in several publications using the EMF induced in the pick-up coil by propagating DW [124,125,126]. When the small pick-up coil is very close to the wire, the time dependence of the EMF induced in the coil can be used to study the details of the moving DW on a length scale comparable to the pick-up coil dimensions. However, the current induced in the coil by traveling DW can affect the DW velocity through a local magnetic field generated in the coil. Therefore, very small coils in the form of a single loop [124] or with a maximum of 10 turns [125] were used to evaluate the DW shape using the EMF signal, ε.

**Figure 33:**
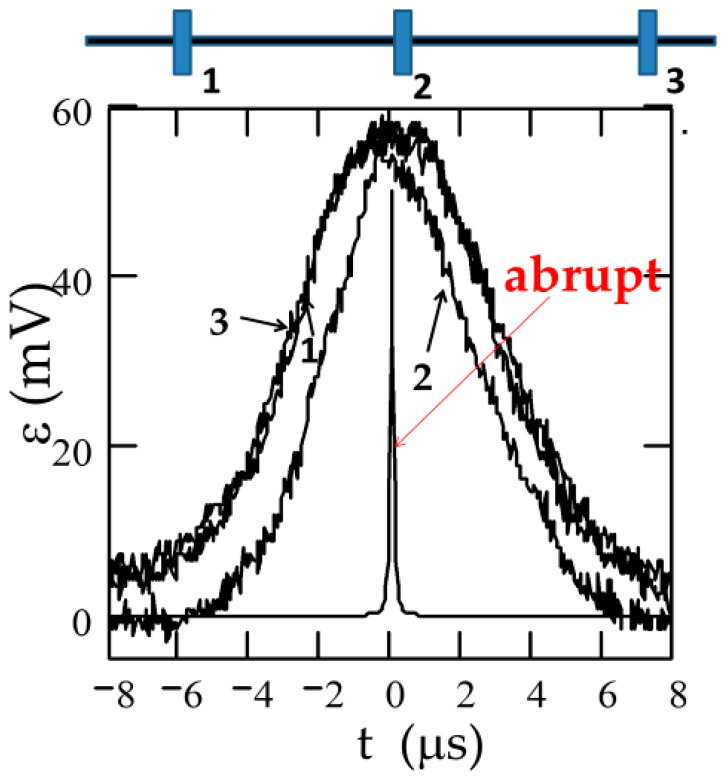
The three EMF peaks from 3 pick-up coils: left (1), center (2), right (3) in Fe_77.5_Si_7.5_B_15_ microwire and the EMF peak from abrupt DW. Adapted from ref. [124].

The EMF peaks recorded by the pick-up coils wound around Fe_77.5_Si_7.5_B_15_ microwire (*ρ* = 0.3) are rather wide (as compared to those calculated from an abrupt DW), which is explained by the fact that the DW is not abrupt (see Figure 33) [124]. Accordingly, such a wide DW would severely limit the information density on the microwire that can be successfully interrogated.

Additionally, the EMF peak from the central pick-up coil (marked as 2) is substantially sharper and narrower than the EMF peaks, ε, from the two end pick-up coils (see Figure 33) [124]. Similarly, different EMF peaks have been observed in other Fe-rich microwires (Fe_74_Si_11_B_13_C_2_ with *ρ* = 0.76) [125].

The common feature of the two wider EMF peaks in the end pick-up coils is that they are generated by the DW between short and long domains. The sharper peak is generated by the DW between two domains of the same length. This feature has been explained considering the influence of the demagnetizing field, *H_d_*, of the axially magnetized core. Accordingly, it was considered that the magnetic field acting on DW during the remagnetization process changes as *H_d_* is affected by the dimensions of two inner domains with opposite magnetization orientation during DW propagation along the wire [124].

Similarly, essentially not abrupt DWs are observed in other Fe-rich microwires, i.e., in Fe_74_Si_11_B_13_C_2_ microwire (*ρ* = 0.62, 0.62, 0.68, 0.76) [125].

**Figure 34:**
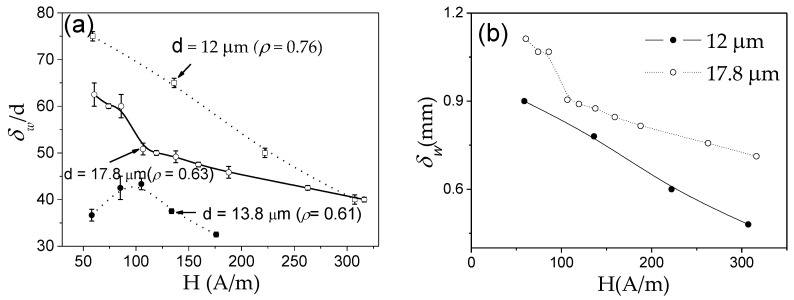
Dependence of characteristic DW width to microwire diameter, d, ratio, *δ_w_*/d on magnetic field, H, (**a**) and dependence of characteristic DW width, *δ_w_*, on magnetic field, H, (**b**) evaluated for Fe_74_Si_11_B_13_C_2_ microwires with different d-values: d = 12 μm, *ρ* = 0.76 d = 13.8 μm, *ρ* = 0.61 and d = 17.8 μm, *ρ* = 0.63. Figure 34a is adapted from ref. [125].

The DW width and its correlation with the microwire diameter, d, and magnetic anisotropy are experimentally and theoretically discussed elsewhere [125]. Thus, in contrast to nanowires where the characteristic DW width, *δ_w_*, is of the order of the wire diameter, i.e., *δ_w_*/d~1–2 [127], in several Fe_74_Si_11_B_13_C_2_ microwires the EMF peaks fit better to *δ_w_*/d~35–75 (see Figure 34a). Such difference is explained considering that the contribution of the exchange energy decreases with the rising of d. However, *δ_w_*/d-ratio is affected by the value of the magnetic anisotropy constant *K*: *δ_w_*/d changes from 13.5 for *K* = 10^4^ erg/cm^3^ up to *δ _w_*/d = 40–50 for *K* = 10^3^ erg/cm^3^, respectively. Additionally, *δ_w_*/d is affected by the applied magnetic field (see Figure 34a,b). From Figure 34b can be evaluated that the DW width can achieve up to 1.2 mm (for Fe_74_Si_11_B_13_C_2_ sample with d = 17.8 μm, *ρ* = 0.63). This *δ_w_* magnitude looks reasonable as for Fe-rich microwire with d ≈ 10 µm the magnetic bistability is observed starting from 2 mm long samples [128].

Similar *δ_w_*-value have been obtained from the EMF peak width, Δ*t*, and measured DW velocity, *v*, considering the uniform DW propagation without oscillation [129]:

*δ_w_* = *v* Δ*t* (10)

**Figure 35:**
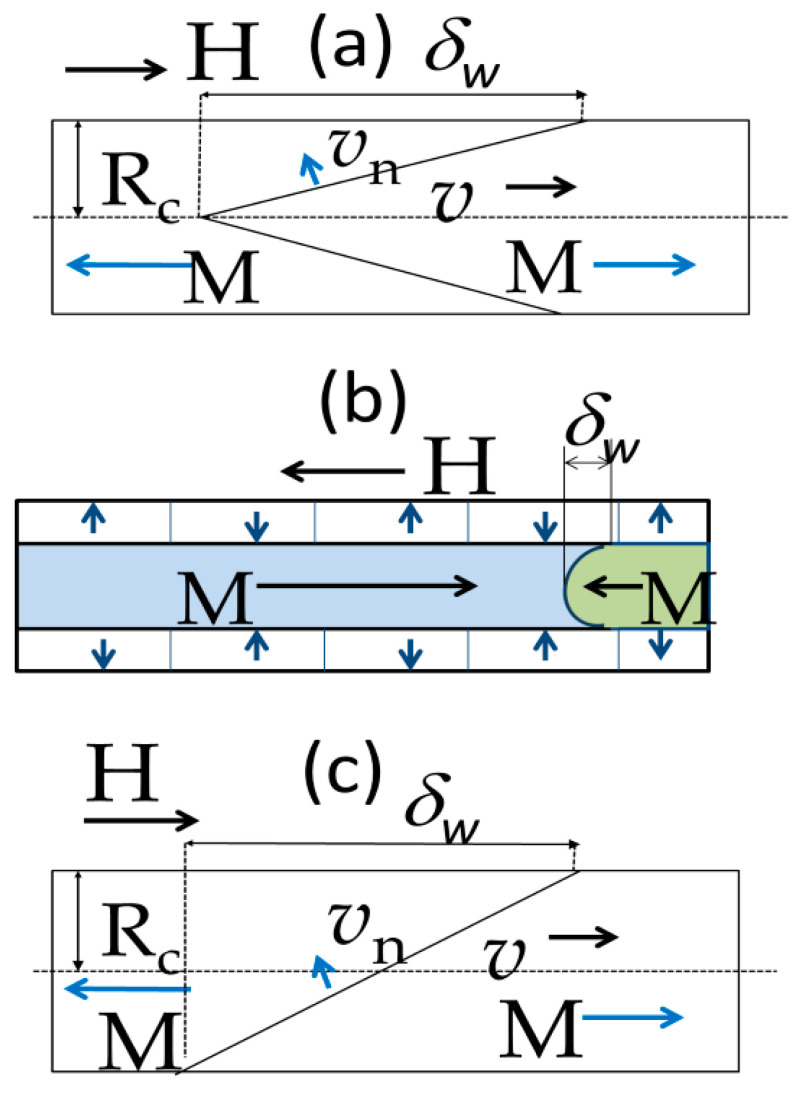
Domain wall shapes: narrow tail at one end close to cylindrical at the other end (**a**), conical (**b**) and planar (**c**) considered for explanation of fast DW dynamics in magnetic microwires.

Several attempts to evaluate the DW shape from the EMF peaks assuming that the DW has a cylindrical symmetry have been made [27,129,130]. The DW shape with a narrow tail at one end close to cylindrical at the other end has been proposed by integrating the EMF peak, ε [129]. Such DW shape reduces the magnetostatic energy by minimizing the surface area (see Figure 35a). However, conical [27,130] and even planar DW shape [126] have been considered (see Figure 35b,c).

The common feature of all considered cases is that the DW between the two domains with the opposite magnetization propagates along the normal vector to the DW surface with a normal velocity related to the axial velocity by a factor *R_c_*/*δ_w_* and could be up to 160 times lower than axial DW velocity, *v* (considering *δ_w_*/d shown in Figure 34a).

From the results provided in Figure 34, it is clear that the DW features, that is, *δ_w_*, can be tuned by *d*, as well as by *K*.

On the other hand, the magnetic anisotropy, and its radial distribution can be effectively modified by stress-annealing (Figure 17e).

**Figure 36:**
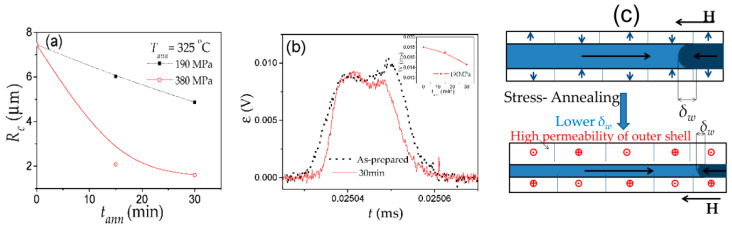
Effect of annealing time, *t_ann_*, at *T_ann_* = 325^o^ C at different stress, *σ_m_* (**a**), EMF peaks induced in pick-up coil measured in as-prepared and stress-annealed (at *σ_appl_* = 190 MPa, *t_ann_* = 30 min) (evolution of the half-width, *W*, of the EMF signal after stress-annealing for magnetic field, *H* = 25 A/m is shown in the inset) (**b**), and schematic picture illustrating the influence of stress-annealing on domain structure and DW width of the microwires (**c**). Adapted from ref. [27].

A decrease in *R_c_* observed after stress-annealing (see Figure 17e and Figure 36a) must be attributed to an increase in the volume of a microwire with transverse magnetic at expense of the inner axially magnetized core with an increase in *σ_m_* (Figure 16e) or *t_ann_* (Figure 36a). Such transformation of domain structure is confirmed by significant GMI effect improvement and magnetic softening of stress-annealed Fe-rich microwires [27,56,96]. Consequently, the radius of the inner axially magnetized core can be tuned by stress-annealing. Accordingly, a decrease in the EMF half-width (full width at half maximum), *W*, signal recorded by the pick-up coil is observed after stress-annealing (see Figure 36b).

Such *W* decrease can be associated either with the decrease of *δ_w_* or increase in *v* [27]. However, careful analysis of both factors considering the change in *v* and remanent magnetization [27] allowed to separate both contributions and conclude that observed evolution of *W* cannot be explained without contribution in the characteristic DW width reduction, as schematically shown in Figure 36c. Consequently, stress-annealing allows remarkable improvement of *S* and *v* because of induced transverse magnetic anisotropy and simultaneously decrease in *δ_w_* (see Figure 36c).

The performance of prospective devices utilizing DW propagation is determined by the degree to which the DW propagation can be controlled, i.e., DW injection, propagation, pinning, and interaction [9,10,131]. Several techniques, like DW injection by local magnetic field or creation of artificial defects allowing either DW pinning or nucleation are proposed [10,56,131].

The creation of structural features such as notches or protrusions patterned in nanowires together with a combination of axial or transverse magnetic fields is demonstrated as an effective method for either creation of multiple domains or DW pinning [131].

In magnetic microwires, an alternative method is proposed, which consists of local (about 2 mm) heating at a selected location of the microwire subjected to tensile stress [56]. This method is based on the remarkable dependence of the stress-annealing induced magnetic anisotropy on *T_ann_*, which allows the creation of a graded magnetic anisotropy using a furnace with variable temperature [56].

The microwire annealed at variable temperature presents rather different hysteresis loops and, hence, graded magnetic properties: different *H_c_* and *M_r_*/*M_s_* along the microwire [56].

Accordingly, in a microwire subjected to local stress-annealing, heated locally (about 2 mm) at a selected place, we created an artificial source of domain wall injection allowing the manipulation of domain wall dynamics.

**Figure 37:**
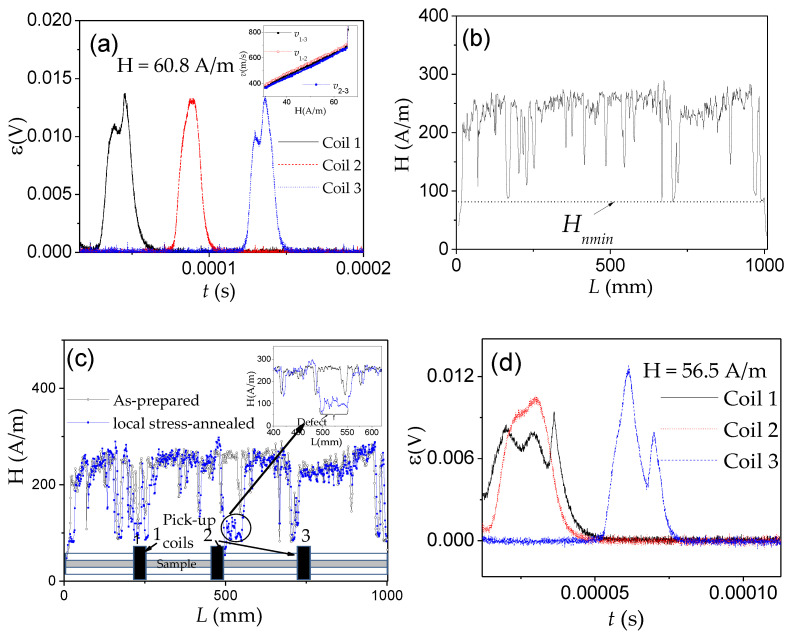
EMF peaks induced by the magnetization change in the pick-up coils (dependencies of DW velocity, *v*, on the magnetic field, H, are shown in the inset) (**a**) local nucleation fields distribution, *H_n_*(*L*), (**b**) of as-prepared Fe_75_B_9_Si_12_C_4_ (*ρ* = 0.88) microwire, local nucleation fields distribution, *H_n_*(*L*), (with zoomed local defect area) (**c**) and *EMF* peaks induced by the magnetization change in the pick-up coils (**d**) of Fe_75_B_9_Si_12_C_4_ (*ρ* = 0.88) microwire subjected to local stress-annealing. Adapted from ref. [56].

The difference in the DW dynamics in as-prepared and locally annealed under tensile stress Fe_75_B_9_Si_12_C_4_ (*ρ* = 0.88) microwires are evidenced in Figure 37. In the as-prepared sample, the moving DW sequentially passes through the first, second and third receiving coils, which indicates a single DW propagation regime, characterized by a linear *v*(*H*) dependence (shown in the inset) for the field range 30 ≤ *H* ≤ 75 A/m (see Figure 37a). *H_nmin_ ≈* 78 A/m, and the nucleation field fluctuations related to the macroscopic inhomogeneities (defects) are typical for *H_n_*(*L*) profiles (as can be compared to Figure 3, Figure 4 and Figure 5) and consistent with linear *v*(*H*) dependence for 30 ≤ H ≤ 75 A/m.

The region with lower *H_n_*-values can be found in the middle part of the locally stress-annealed sample from the comparison of *H_n_*(*L*) profiles of as-prepared and locally stress-annealed microwires (see Figure 37c).

Accordingly, the sequence of the *EMF* peaks in the locally stress-annealed sample has been changed: the peaks in pick-up coil 1 and coil 2 appear almost simultaneously (Figure 37c). This means that the DW passes almost simultaneously through coils 1 and 2. Consequently, the simultaneous propagation of two DWs (the first DW moves from the sample end, as it was observed for the as-prepared microwire, and the second DW is generated at a local defect created by local stress-annealing) should be assumed for locally stress-annealed microwires.

As shown above (Figure 3, Figure 4, and Figure 37c), *H_n_*-values near the sample ends are typically considerably lower than in the middle part of each as-prepared microwire. Thus, DWs usually depin from the end closure domains at the microwire even in a weak magnetic field. The described above method allows the creation of the region in the middle part of the microwire with a low *H_n_*-value. Accordingly, magnetic-field-driven DW propagation can start from a place with an even lower *H_n_*-value.

DW dynamics in microwires can also be tuned using the magnetostatic interaction between microwires, as described in [132]. The hysteresis loop of an array containing two Fe-based closely placed microwires consists of two Barkhausen jumps, being rather different from that of a single Fe-rich microwire [132]. Such peculiar hysteresis loop shape has been explained considering the magnetostatic interaction originated by stray fields created by magnetically bistable microwires. In such a microwire array, the DW starts to move at a lower applied field and the domain wall velocity is higher than in the single microwire case. The neighboring microwire serves as a source of the additional magnetic field and in the case of the first Barkhausen jump, it promotes remagnetization of the affected microwire, effectively decreases the switching field value, and increases the domain wall velocity [132].

**Figure 38:**
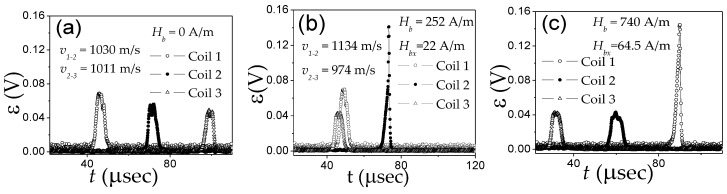
Voltage peaks induced by the propagating DW in the 3 pick-up coils in Fe_74_B_13_Si_11_C_2_ (*ρ* ≈ 0.67) microwires measured at H = 140 A/m with different values of bias magnetic field, *H_b_*, and axial projection *H_bx_*: *H_b_* = 0 A/m (**a**) *H_b_* = 252 A/m(**b**) *H_b_* = 740 A/m(**c**). Reprinted with permission from ref. [57].

Another possibility to control the DWs dynamics in microwires is the engineering of collisions between two moving DWs in magnetic microwires. Such DW collision has been observed in magnetic microwires when DW propagation from the opposite microwire end has been activated by applying the bias sketched field, *H_b_*, at different angles with respect to the wire axis [57]. The DW collision can be verified from the *ε*(*t*) dependencies recorded at different *H_b_* shown in Figure 38b,c. As can be appreciated from Figure 38a–c, the DW collisions between two moving DWs can be observed in different places of the magnetic microwire by varying *H_b_*. Thus, at certain *H_b_* (*H_b_* ≈ 252 A/m), a DW collision can be observed near the position of the 2nd coil, as evidenced by a drastic increase in the signal height from coil 2 (Figure 38b). Additionally, at a further increase in *H_b_*, the DW from the opposite microwire arrives at coil 1 simultaneously with the DW propagating from the nearest microwire end (Figure 38c), and hence the DW collision is observed by coil 1 (Figure 38c). Consequently, by controlling the bias field we can release the DWs at targeted locations in the microwire.

The other example of controllable DW propagation in microwires is the ordered motion of several DWs, either in unison or in opposite directions, leading to the annihilation of DWs by combining opposing effects of sets of local-coil fields distributed along a microwire [133].

The DW features in magnetic microwires are rather different from those in nanowires or thin films. As discussed above, in contrast to nanowires where *δ_w_*/d~1–2, in amorphous microwires *δ_w_*/d~35–75. Therefore, the nucleation of DWs at defects rather than DW pinning is observed in magnetic microwires. In contrast, in thin films, the efficient domain-wall pinning can be engineered by growing ultra-thin magnetic films with perpendicular anisotropy on a patterned substrate exhibiting sub-nanometer steps modulation [134]. The above-provided solutions, allow either annihilation of DWs nucleated on defects or the controlled nucleation of DWs on artificially created inhomogeneities. However, a traveling DW can be braked and even trapped in a given position when a local antiparallel magnetic field is applied. In addition, the position of the DW and its velocity can be controlled by a suitable setting of the local magnetic field [55].

Although in most publications on magnetization reversal of magnetically bistable microwires, the origin of perfectly rectangular hysteresis loops is attributed to fast DW propagation along the microwire [5,16,34], such a mechanism is not unique. Perfectly rectangular hysteresis loops (see for example Figure 15) can be also attributed to the magnetization reversal by coherent rotation in single-domain magnets [135,136]. The model describing the coherent magnetization rotation assumes that all spins in the system are parallel with respect to each other, at any time. However, as the size of the magnet is increased, the magnetostatic energy in such a system where all the spins are parallel becomes high. As a result, magnetic domains are formed, resulting in a minimized energy state. On the other hand, when the magnets are very small (i.e., magnetic nanoparticles) magnetostatic energy is not high. Coherent reversal of magnetization can be realized in such nanostructures. In the present case, closure domains at the microwire ends have been observed by several methods [128]. Additionally, the DW propagation, the shape and characteristic width of traveling DWs have been evaluated by several methods [124,125]. Therefore, DW depinning of a DW from the closure end domains with its subsequent propagation is commonly considered as a fast magnetization switching mechanism [5,34,35,36].

On the other hand, by manipulating the microwire composition, the saturation magnetization, and, thus, the shape anisotropy field could be substantially reduced. Similarly, the transverse magnetic anisotropy can reduce the magnetization reversal field by coherent magnetization rotation. Additionally, the diameter of magnetic wires prepared using the Taylor–Ulitovsky method can be substantially reduced [24]. Therefore, we cannot exclude that the magnetization reversal by coherent rotation can be observed either in thinner amorphous nanowires or upon application of the pulsed reversed magnetic field.

Accordingly, the aforementioned overview of the recent studies on DW propagation in micrometric and submicrometric wires enabled us to identify several routes to engineer the DW dynamics, as given below:

The DW velocity and mobility can be improved by minimizing the magnetoelastic anisotropy, either through the selection of an amorphous alloy with a low magnetostriction coefficient or by appropriate annealing of microwires with spontaneous magnetic anisotropy to facilitate the internal stress relaxation.

- Appropriate annealing can be used to produce magnetically bistable microwires from initially non-magnetically bistable microwires. These magnetically bistable microwires can exhibit DW dynamics with an unusual stress.
- The most appropriate magnetic anisotropy distribution can be designed using post-processing (i.e., by inducing magnetic anisotropy and design of its radial distribution).
- The amorphous matrix can be devitrified, and as-prepared microwires with nanocrystalline structures can be fabricated.
- The chemical composition of microwires with high saturation magnetization can be beneficial for DW dynamics improvement.
- Technology improvement can be used to decrease the defect content and hence extend the single DW propagation regimes.

Although the DWs of amorphous microwires are generally not abrupt and exhibit a complex shape, the DW characteristic width can be tuned either by selecting the microwire diameter or even inducing magnetic anisotropy.

Several routes of DW manipulation using a local magnetic field, or artificially created defects as means of DW injection or DW collision have been proposed for DW annihilation.

Amorphous and nanocrystalline microwires provide a unique means of experimentally studying the influence of external stimuli on single DW propagation, such as applied or internal stresses, transverse and local magnetic fields, or intrinsic properties, such as the magnetostriction coefficient, as well as magnetoelastic and induced magnetic anisotropies.

Accordingly, amorphous microwires with excellent mechanical and corrosive properties and extremely high DW velocities can be considered for various prospective applications, such as electronic surveillance, magnetic tags, magnetoelastic sensors, and devices, as well as racetrack memories or magnetic logics.

## 4. Conclusions

In this review, routes for tuning the DW dynamics are summarized, correlation of the DW shape and width with the microwire dimensions and, the magnetic anisotropy and its radial distribution are presented, and routes for manipulating DWs in cylindrical micrometric wires are discussed. The factors affecting the features of single DW dynamics in amorphous and nanocrystalline microwires, such as the DW velocity and, mobility and the extension of the linear field dependence of the DW velocity, are thoroughly analyzed. The correlations between the extension of the linear dependence of the magnetic field and the local nucleation field distribution, defects, and routes enabling faster DW dynamics are provided. An overview is presented of the effects of magnetoelastic, induced and magnetocrystalline anisotropies on the DW mobility and DW velocity, as well as how the DW mobility and velocity can be modified by the appropriate post-processing of amorphous and nanocrystalline microwires with spontaneous and annealing-induced magnetic bistability. Optimization of the DW dynamics by minimizing the magnetoelastic anisotropy, either by selecting a chemical composition with a low magnetostriction coefficient or by applying heat treatment to facilitate the internal stress relaxation is demonstrated. Stress-annealing can be used to design a magnetic anisotropy distribution that promotes an increase in the DW velocity in amorphous magnetic microwires. The beneficial optimization of the DW dynamics by stress-annealing is attributed to the induction of transverse magnetic anisotropy, which has a similar effect on the DW dynamics as an applied transverse magnetic field.

The magnetoelastic anisotropy contribution in the DW dynamics is evidenced by the remarkable stress dependence of the DW dynamics in microwires with spontaneous and annealing-induced magnetic bistability. The unusual stress dependence of the DW dynamics in microwires with annealing-induced magnetic bistability is discussed in terms of the stress dependence of the magnetostriction coefficient.

Current-driven DW propagation in microwires with annealing-induced magnetic bistability is explained in terms of the magnetostatic interaction of the outer domain shell with the transverse magnetization orientation and the inner axially magnetized core.

Routes for the DW dynamics optimization are presented for various families of nanocrystalline microwires. The role of saturation magnetization in improving DW mobility is discussed.

The DW shape and its correlation with the magnetic anisotropy constant and the microwire diameter, are discussed, as well as how induced magnetic anisotropy can be used to manipulate the DW shape. The use of local stress annealing and DW collisions to engineer the DW propagation is demonstrated.

Properly processed amorphous microwires can present extremely high DW velocity and mobility. In addition to unusually high DW velocity values, amorphous microwires present a combination of superior mechanical and anti-corrosive properties, making them suitable for technical applications. The combination of the unusually fast DW dynamics with excellent mechanical and anti-corrosion properties makes amorphous microwires suitable for a variety of technical applications. However, DWs of amorphous microwires are essentially not abrupt. Several routes, such as induced transverse magnetic anisotropy and defects monitoring, can serve for further DW dynamics improvement in amorphous and nanocrystalline microwires.

## Figures and Tables

**Table 1:** Compositions, geometry, and magnetostriction coefficient of studied glass-coated microwires.

Composition	Metallic Nucleus Diameter, d (μm)	Total Diameter, D (μm)	Ratio *ρ* = d/D	Magnetostriction Coefficient, *λ_s_* × 10^−6^
Fe_77.5_Si_7.5_B_15_	15.1	35.8	0.42	38
Fe_77.5_Si_7.5_B_15_	12	40	0.3	38
Fe_75_B_9_Si_12_C_4_	15.2	17.2	0.88	38
Fe_75_B_9_Si_12_C_4_	13.6	16	0.85	38
Fe_74_Si_11_B_13_C_2_	12	15.8	0.76	38
Fe_74_Si_11_B_13_C_2_	13.8	22.3	0.62	38
Fe_74_Si_11_B_13_C_2_	17.8	28.3	0.63	38
Fe_74_Si_11_B_13_C_2_	16.2	23.8	0.68	38
Fe_74_Si_11_B_13_C_2_	14.6	21.8	0.55	38
Fe_62_Ni_15.5_Si_7.5_B_15_	14.35	33.25	0.43	27
Fe_49.6_Ni_27.9_Si_7.5_B_15_	14.2	33.85	0.42	20
Fe_47.4_Ni_26.6_Si_11_B_13_C_2_	29	32.2	0.9	20
Co_69.2_Fe_3.6_Ni_1_B_12.5_Si_11_Mo_1.5_C_1.2_	22.8	23.2	0.98	−1
Co_69.2_Fe_4.1_B_11.8_Si_13.8_C_1.1_	25.6	30.2	0.85	−0.03
Fe_71.8_Cu_1_Nb_3.1_Si_15_B_9.1_	6.6	18.4	0.36	30
Fe_70.8_Cu_1_Nb_3.1_Si_14.5_B_10.6_	5.8	15.2	0.38	30
Fe_38.5_Co_38.5_B_18_Mo_4_Cu_1_	10	16.6	0.6	30
Fe_73.5_Cu_1_Nb_3_Si_11.5_B_11_	10	28	0.36	30
Fe_8.1_Co_50.7_Ni_17.6_B_13.3_Si_10.3_	11.6	14	0.83	−0.9
Co_56_Fe_8_Ni_10_Si_10_B_16_	22	26.2	0.84	0.1
Fe_66.7_Cr_11.4_B_12_Si_9_Ni_0.9_	17.6	38.6	0.46	7
Co_41.7_Fe_36.4_Si_10.1_B_11.8_	13.6	24	0.57	25
Co_41.7_Fe_36.4_Si_10.1_B_11.8_	13.6	34	0.4	25
Co_41.7_Fe_36.4_Si_10.1_B_11.8_	18	38	0.47	25
Fe_16_Co_60_Si_13_B_11_	12	29	0.41	15
Fe_55_Co_23_B_11,8_Si_10.2_	13.2	29.6	0.45	30
Fe_38.5_Co_38.5_B_18_Mo_4_Cu_1_	9.4	22.5	0.41	
Fe_38.5_Co_38.5_B_18_Mo_4_Cu_1_	10	16.6	0.6	
(Fe_0.7_Co_0.3_)_83.7_Si_4_B_8_P_3.6_Cu_0.7_	15.5	26.5	0.58	
